# Concurrent Metabolic Profiling and Quantification of Aromatic Amino Acids and Phytohormones in *Solanum lycopersicum* Plants Responding to *Phytophthora capsici*

**DOI:** 10.3390/metabo10110466

**Published:** 2020-11-16

**Authors:** Msizi I. Mhlongo, Lizelle A. Piater, Paul A. Steenkamp, Nico Labuschagne, Ian A. Dubery

**Affiliations:** 1 Department of Biochemistry, University of Johannesburg, P.O. Box 524, Auckland Park, Johannesburg 2006, South Africa; mmhlongo@uj.ac.za (M.I.M.); lpiater@uj.ac.za (L.A.P.); psteenkamp@uj.ac.za (P.A.S.); 2 Department of Plant and Soil Sciences, University of Pretoria, Private Bag X20, Hatfield, Pretoria 0028, South Africa; nico.labuschagne@up.ac.za

**Keywords:** aromatic amino acids, correlation, metabolomics, *Phytophthora capsici*, phytohormones, tomato, time-dependent metabolic reprogramming

## Abstract

Pathogenic microorganisms account for large production losses in the agricultural sector. *Phytophthora capsici* is an oomycete that causes blight and fruit rot in important crops, especially those in the Solanaceae family. *P. capsici* infection is difficult to control due to genetic diversity, arising from sexual reproduction, and resistant spores that remain dormant in soil. In this study, the metabolomics of tomato plants responding to infection by *P. capsici* were investigated. Non-targeted metabolomics, based on liquid chromatography coupled to mass spectrometry (LC-MS), were used with multivariate data analyses to investigate time-dependent metabolic reprogramming in the roots, stems, and leaves of stem-infected plants, over an 8 day period. In addition, phytohormones and amino acids were determined using quantitative LC-MS. Methyl salicylate and 1-aminocyclopropane-1-carboxylate were detected as major signalling molecules in the defensive response to *P. capsici*. As aromatic amino acid precursors of secondary metabolic pathways, both phenylalanine and tryptophan showed a continuous increase over time in all tissues, whereas tyrosine peaked at day 4. Non-targeted metabolomic analysis revealed phenylpropanoids, benzoic acids, glycoalkaloids, flavonoids, amino acids, organic acids, and fatty acids as the major classes of reprogrammed metabolites. Correlation analysis showed that metabolites derived from the same pathway, or synthesised by different pathways, could either have a positive or negative correlation. Furthermore, roots, stems, and leaves showed contrasting time-dependent metabolic reprogramming, possibly related to the biotrophic vs. necrotrophic life-stages of the pathogen, and overlapping biotic and abiotic stress signaling. As such, the targeted and untargeted approaches complemented each other, to provide a detailed view of key time-dependent metabolic changes, occurring in both the asymptomatic and symptomatic stages of infection.

## 1. Introduction

*Phytophthora capsici* is an oomycete pathogen with a broad host range, and regarded as one of the 10 most important plant pathogens. *P. capsici* infects more than 45 plant species (e.g., cucurbits, pepper, eggplant, and tomato, etc.), causing damage (crow-, root-, and fruit-rot) to many economically important crops. In this regard, disease occurrence and severity has escalated in recent years, leading to economic losses worldwide. *P. capsici* is a hemibiotrophic pathogen, with an initial biotrophic phase followed by a switch to a necrotrophic lifestyle. Depending on environmental conditions, infection of tomato plants can occur via the roots, crown and foliage, or even the fruit [1,2,3].

In general, plants are continuously exposed to attack by pathogenic microorganisms, both above and below ground. In defence, multi-layered mechanisms consisting of constitutive and inducible responses are employed [4,5]. The initial stages of a plant–pathogen interaction include the perception of microbe/pathogen-associated molecular patterns (M/PAMPs) or damage-associated molecular patterns (DAMPs) by specialised pattern recognition receptors (PRRs) [6,7,8], activation of intracellular signalling (mostly the mitogen-activated protein kinase (MAPK) pathway), generation of reactive oxygen species (ROS), and production of defence-associated phytohormones [9,10,11,12]. The latter play a pivotal role in plant biochemical processes, including defence responses [4,13,14], and accumulate in varying amounts in response to attempted infection or insect attacks, thereby leading to reprogramming of the transcriptome, activation of defence genes, and production of phytoalexins [4,15,16]. To minimise the fitness costs of unnecessary activation of defence genes, and to launch a specific immune response, plants produce a mixture of hormones that are specific to the stress detected. Salicylic acid (SA)-induced resistance is more effective against biotrophic pathogens [17], while jasmonic acid (JA)- and/or ethylene (ET)-induced resistance is operative against necrotrophic pathogens and herbivore attack. Different plant species can employ different signal transduction pathways to specify the immune response. Other phytohormones, including cytokinins, auxins, abscisic acid (ABA), gibberellins, and brassinosteroids, have been reported to play a role in plant resistance; however, knowledge regarding the significance of these molecules is limited [4,14,18,19]. These hormones interact either antagonistically or synergistically with the SA-JA-ET signalling backbone, and reprogram the defence output [19,20,21].

Following perception and signalling, plants modify their cell walls through lignin synthesis and callose deposition, as well as the biosynthesis of antimicrobial phytoalexins [4,22,23]. The ability of plants to mount an immune response under stress conditions is associated with a high probability of survival under such conditions. However, these responses are energy demanding and have high fitness costs. For plant defence execution, energy is critical, and plays an important role in defence gene expression in support of various metabolic pathways [24]. Thus in the past, attempts have been made to understand the underlying metabolic pathways involved in plant physiological processes (energy regulation, growth and development of tissue, and reproduction) and defence response regulation [25]. Amino acids are molecules used as sources of carbon and nitrogen, and are precursors of the molecular skeletons of compounds to be synthesised. Upon infection, synthesis of these metabolites can be supported by energy-generating pathways such as the tricarboxylic acid (TCA) pathway, glycolysis, and the pentose phosphate pathway [25,26], which then drives immune defence responses. The 20 amino acids can be converted by various enzymatic reactions to seven precursors and intermediates (α-ketoglutarate, acetoacetate, acetyl-CoA, fumarate, oxaloacetate, pyruvate, and succinyl-CoA) for energy generation [27]. In turn, the aromatic amino acids (Phe and Tyr) provide the phenolic group for the production of various phenolic molecules via the phenylpropanoid pathway, and these molecules have direct and indirect effects on pathogens [28,29,30,31]. Aromatic amino acids (Trp and Tyr) can also be converted to various amines such as serotonin and tyramine [32]. Trp also serves as a precursor for the production of indole-3-acetic acid, a phytohormone playing a pivotal role in plant growth and resistance [33,34].

Under stressed conditions plants are known to sacrifice some energy required for physiological processes, which is then channelled towards defence activation [27,35], and is associated with primary metabolite modification. Using metabolomics, one can obtain a “snapshot-in-time” of these events, and to deduce metabolic reprogramming taking place during plant defence responses. By definition, this omics approach is an unbiased analysis of the whole metabolome in a living system [36]. However, chemical diversity of metabolites is a major challenge, making whole metabolome analysis difficult [36,37]. Depending on the chosen analytical platform, the extraction method will favour certain metabolites in the said scenario.

*P. capsici* is a broad-host-range pathogen, and disease initiation and progression will depend on the site of infection, the initial inoculum, the physiological condition of the plants, and the inherent ability of the cultivar to withstand the infection/counter the actions of the pathogen [1,2,3]. Previous studies have investigated plant defences to *P. capsici,* with no/very few metabolic studies that included looking at the metabolic reconfiguration correlated to the defensive state of the plant to the pathogen. Thus, in the present study, both non-targeted and targeted analyses were used to investigate the time-dependent metabolic reprogramming in tomato plants responding to *P. capsici* infection.

## 2. Results

### 2.1. Symptom Development

At the outset, we monitored the symptom progression in the tomato plants (*S. lycopersicum* “Moneymaker”) inoculated with *P. capsici* spores in time-course experiments (Figure S1). As observed in previous studies [38], disease symptoms were observed 4 to 8 d post-inoculation (d.p.i.), with day 8 showing a complete diseased state. Symptoms included wilting and crown rot, and, in some instances, the lower stem collapsed (stem rot due to stem inoculation), leading to rapid wilting of the canopy and plant death (Figure S2). *Phytophthora* spp. are destructive pathogens with a hemi-biotrophic lifestyle (this features a biotrophic phase which then switches to a necrotropic phase) [39]. Thus, *P. capsici* infection involves a biotrophic phase in which the host plant appears healthy and unharmed, lasting for 0–2 d (Figure S1). Following successful infection, the pathogen switches to the necrotrophic phase (4–8 d), accompanied by wilting and subsequent plant collapse (Figure S1).

### 2.2. Aromatic Amino Acid Quantification

**Figure 1:**
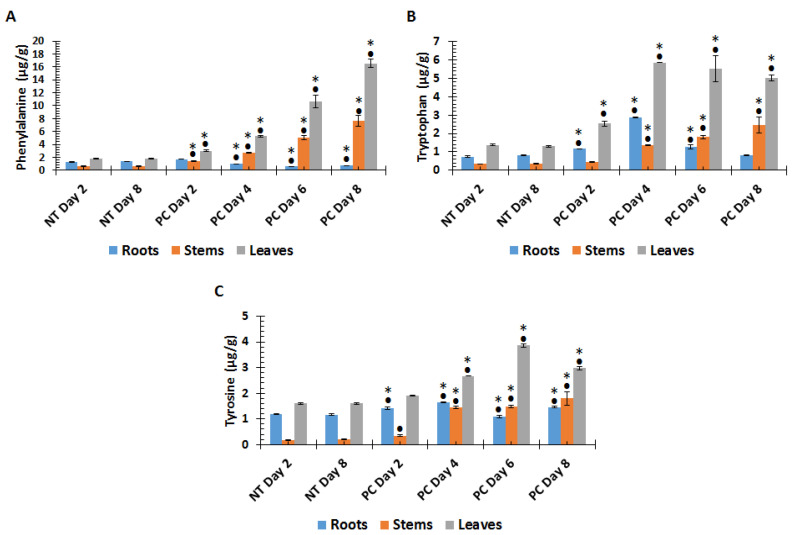
Changes in aromatic amino acid content in tomato plant tissue harvested at different time points following *Phytophthora capsici* (PC) inoculation in stems vs. non-treated (NT) plants. (**A**): Phenylalanine, (**B**): Tryptophan, (**C**): Tyrosine. Upon harvesting, the plants were divided into root, stem, and leaf tissue. Values are means ± SD (*n* = 3 independent sampling). Extracts were prepared from 200 mg of pulverised tissue, and all concentrations are expressed as µg/g fresh weight (FW). An asterisk (*) or a dot (•) indicates the statistical significance with a *p*-value < 0.05 compared with the non-treated plants, with • indicating a comparison between NT day 2 and the PC infection on days 2–8 post-infection, and * a comparison between NT day 8 and the PC infection on days 2–8 post-infection. The results show a time-dependent differential reprogramming of aromatic amino acids levels in the various tissues.

In order to investigate the involvement of aromatic amino acids (Phe, Trp, and Tyr) in the tomato plant response to *P. capsici* inoculation, 50% methanol extracts of roots, stems, and leaves were analysed using an multiple reaction monitoring (MRM) method, based on ultra-high-performance liquid chromatography triple quadrupole mass spectrometry (UHPLC-QqQ-MS). The content of Phe (Figure 1A) was found to be differentially modulated by *P. capsici* inoculation, where significantly increased accumulation in stems (1.8–8 µg/g) and leaves (3.8–17 µg/g) from 2 to 8 d.p.i. were found, while a decrease (1.5–0.4 µg/g) over the same time period was observed in the roots when compared to non-treated (NT) plants. The Trp (Figure 1B) content followed the same trend as Phe (accumulation from day 2 to 8) in both stems and leaves of infected plants, with a range increase of 1.3–2.5 µg/g and 2.5–5.5 µg/g, respectively. However, the content in roots increased from 1.3–3.2 µg/µg between day 2 and 4 post-inoculation, and then decreased to ~1.3 µg/g thereafter (day 6 and 8) (Figure 1B). Lastly, the quantities of Tyr increased in both stems and leaves (similar trend to Phe and Trp) (Figure 1C), from 0.1–1.8 µg/g and 1.5–3.8 µg/g, from 2 to 8 d.p.i., respectively. However, in leaves, a slight decrease from 3.8–3.0 µg/g was observed on day 8 (Figure 1C). In roots, the Tyr content in *P. capsici*-treated plants increased from days 2 to 4, followed by a slight decrease on days 6 to 8, but remained significantly higher than that of the NT plants (Figure 1C).

### 2.3. Quantification of Methyl Salicylic Acid and 1-Aminocyclopropane-1-Carboxylic Acid

**Figure 2:**
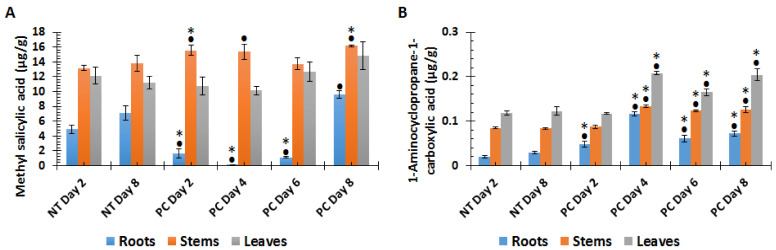
Changes in phytohormone levels in tomato plant tissues harvested at different time points following *P. capsici* (PC) inoculation in stems vs. non-treated (NT) plants. (**A**): Methyl salicylic acid and (**B**): 1-Aminocyclopropane-1-carboxylic acid. Extracts were prepared from 200 mg of pulverised tissue, and all concentrations are expressed as µg/g fresh weight (FW). An asterisk (*) or a dot (•) indicates the statistical significance with a *p*-value < 0.05 compared with the non-treated plants, with • indicating a comparison between NT day 2 and the PC infection on days 2–8 post-infection, and * a comparison between NT day 8 and the PC infection on days 2–8 post-infection. The results show a time-dependent differential reprogramming of MeSA and ACC in the various tissues.

Six phytohormones (SA and methyl salicylate, MeSA; indole acetic acid, IAA; ABA; JA, and methyl jasmonate, MeJA) as well as the ET precursor, 1-aminocyclopropane-1-carboxylic acid (ACC), were targeted for quantification. However, only two (MeSA and ACC) are presented here, as they showed differential regulation during pathogen progression, and were found to be above the LOQ for the different time points. In root tissues, the MeSA content significantly decreased with time, followed by a sharp increase on day 8 (PC day 8) (Figure 2A). In stem tissue, the MeSA content was significantly higher than both controls on days 2 and 8, whereas at day 4 a significant difference was observed between NT day 2 and PC day 4 plants. On day 6 no significant difference was observed when compared to both controls (NT day 2 and NT day 8) (Figure 2A). The ACC content showed increased accumulation in roots from day 2 to day 4 post-infection, after which a decrease was observed; however, it remained significantly higher than the NT plants over time (Figure 2B). In stems, significantly higher contents of ACC were observed from 4 to 8 d.p.i. (Figure 2B). A similar trend was observed in leaves where the ACC content was significantly higher from 4 to 8 d.p.i. (Figure 2B).

### 2.4. Metabolic Profiling of P. capsici-Induced Changes in Tomato Plants

#### 2.4.1. Multivariate Data Analysis

Various studies have shown that plant–pathogen interactions lead to the activation of different metabolic pathways and the concomitant accumulation of defence metabolites. In order to gather such metabolic information, a non-targeted approach was used to analyse the methanol extracts of non-treated (NT) and *P. capsici* (PC)-infected tomato plants with the aid of ultra-high-performance liquid chromatography, quadrupole time-of-flight mass spectrometry (UHPLC-qTOF-MS) analysis. Plant samples are known to contain thousands of highly diverse molecules (both chemically and structurally diverse); as such it is important to first resolve the sample constituents by chromatographic separation, followed by electrospray ionisation (ESI)-MS to detect the eluents. Base peak intensity (BPI) chromatograms (Figures S3–S5) of the tomato plant extracts following treatment with *P. capsici* showed some variation in peak intensities, accumulation of new peaks, and disappearance of some peaks. This is an indication that *P. capsici* altered cellular metabolism, resulting in time-dependent metabolic changes.

**Figure 3:**
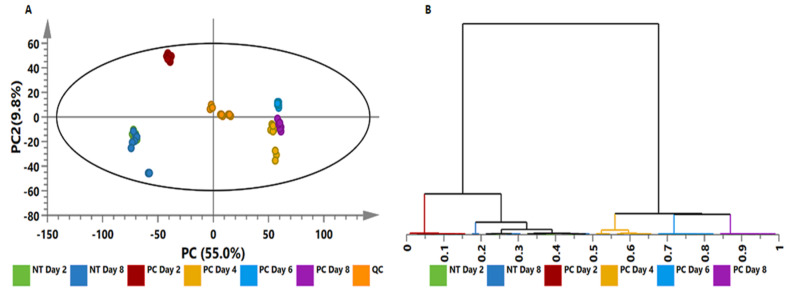
Unsupervised statistical analysis of tomato plants infected with *Phytophthora capsici;* leaf data acquired in electrospray ionisation (ESI^−^) mode. (**A**): A principal component analysis (PCA) scores scatter plot of all the samples, including the quality control (QC) samples, coloured according to time points. The PCA model presented here was a 7-component model, with R^2^ of 0.801 and Q^2^ of 0.737. (**B**): The hierarchical clustering (HC) dendrogram corresponding to (A). Unsupervised statistical analysis was used to generate subgrouping of samples based on similar observations in (A), while the HC dendrogram shows the hierarchical relationship between samples (B). Similar figures (both for ESI^−^ and ESI^+^ modes) were generated for stem and root tissue and are presented as Figures S6A–S10A. NT = non-treated and PC = *P. capsici*-treated/stem inoculated.

Metabolomic analyses generate huge data sets that are difficult to visually explore, and it is difficult to explain all differences observed in an MS chromatogram. As such, statistical analyses are used to mine the collected multidimensional data, and to pinpoint signatory biomarkers that provide valuable biological information [36,40,41]. Principal component analysis (PCA) is a tool used to explore the data (identification of trends and patterns within the data), leading to the generation of predictive models [40,42]. PCA models were computed from both ESI positive and negative data to provide a visual evaluation of the tomato plant tissues (roots, stems, and leaves) responding to *P. capsici* inoculation. This showed time-related clustering of the various samples (Figure 3A and Figures S6A–S10A), equating to metabolic reprogramming. Furthermore, the pooled biological quality control (BPQC) samples clustered in the middle of the PCA scores plot, reflecting the UHPLC-MS stability, reliability, and reproducibility of the data acquired. The PCA-extracted trends and patterns were further examined by hierarchical clustering (HC) analysis to build clusters. The HC models were computed with Ward’s linkage method considering “between” and “within” cluster distances, and the trees were sorted based on size [43,44,45]. The computed HC models displayed time-dependent sub-clustering (Figure 3B and Figures S6B–S10B). Both PC and HC analyses thus revealed the overall structure of the data, showing underlying trends and patterns in the acquired data sets. These observations clearly indicate the time-dependent metabolic reprogramming in the various tomato tissues in response to *P. capsici* inoculation.

**Figure 4:**
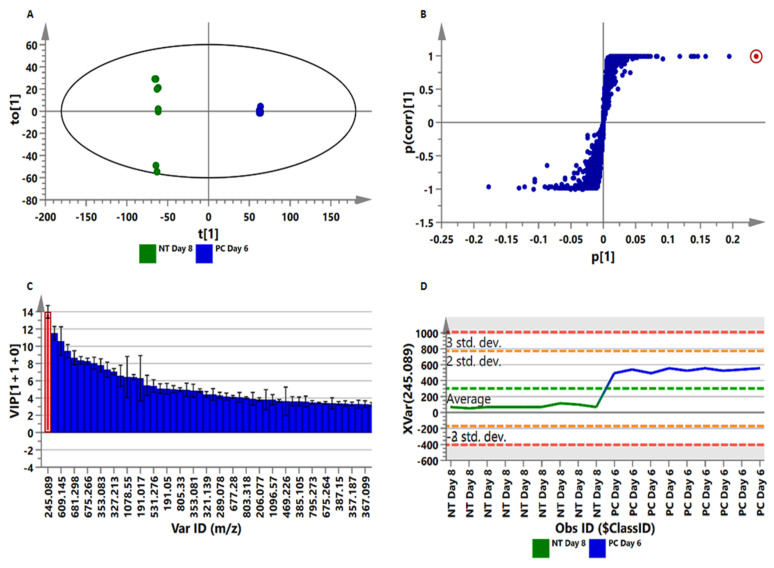
Orthogonal-partial least square discriminant analysis (OPLS-DA) modelling and variable/feature selection of tomato plants infected with *Phytophthora capsici;* leaf data acquired in ESI^−^ mode. (**A**): A typical OPLS-DA score plot separating non-treated (NT) day 8 plants vs. *P. capsici* (PC)-treated day 6 plants (1 + 1 + 0) components, R^2^X = 0.802, Q^2^ = 0.999, CV-ANOVA *p*-value = 1.3 × 10^−^^19^). (**B**): An OPLS-DA loadings S-plot for the same model in A; only variables with the correlation [*(p(corr)*] ≥ |0.6| and covariance *(p1)* ≥ |0.05| were chosen as discriminating variables, and identified using the *m/z* to generate an elemental composition. (**C**): A variable importance in projection (VIP) plot for the same model, pointing mathematically to the importance of each variable in contributing to group separation in the OPLS-DA model. (**D**): A typical variable trend plot (of the selected variable in VIP and S-plots), displaying the changes of the selected variables across the samples (NT day 8 vs. PC day 6). This shows that the selected features significantly discriminated the treated from the control samples. Similar figures (both ESI^−^ and ESI^+^) were generated for stem (site of inoculation) and root tissue, presented as Figures S11–S15.

To complement the exploratory data provided by PCA (Figure 3A and Figures S6A–S10A) and HC analysis (Figure 3B and Figures S6B–S10B) models, a predictive model, orthogonal-partial least square discriminant analysis (OPLS-DA), was used to evaluate and explain the metabolic reprogramming occurring in tomato plants in response to *P. capsici* infection. The computed OPLS-DA models (Figure 4A and Figures S11A–S15A) show a clear separation of control plants (NT day 8) from the treated samples (PC day 6), thus indicating different metabolic profiles. PC day 6 was chosen based on the development of the symptoms; on day 8 the plants were already dying, and to avoid responses not related to the infection, day 6 was chosen. The OPLS-DA models were validated using the R^2^ and Q^2^ metrics, and the analysis of variance testing for cross-validated predictive residuals (CV-ANOVA, *p*-value ˂ 0.05 indicates a good model) [41,46].

For extraction of the signatory biomarkers responsible for the observed sample separation on OPLS-DA, the loadings S-plot (a selection method for variables) (Figure 4B and Figures S11B–S15B) was used for feature selection. To validate the significance of the latter, variable importance in projection (VIP) plots (Figure 4C and Figures S11C–S15C) were used, and variables with a score of >1 were considered as significant (and subsequently selected for compound annotation). VIP plot evaluation prevents bias in the selection of variables, and helps to describe the importance of the variables to the model [41]. Lastly, a variable trend plot (Figure 4D and Figures S11D–S15D) of the S-plot and VIP plot selected variable (highlighted in red) was computed to evaluate the change of the selected feature across the samples (NT vs. PC). Various models (both PCA and OPLS-DA), comparing the metabolomes from different days (PC day 2–8) against controls (NT days 2 and 8) were generated. Due to the large number of graphs generated, not all can be shown. The features found to distinguish NT from PC were then selected for compound annotation. Owing to the lack of commercially available authentic standards, annotations represent putative identifications with assigned features at a metabolite identification (MSI) level-2 annotation [47,48], and are summarised in Table S1, according to retention time (Rt), ESI mode, *m*/*z*, empirical formula, and diagnostic fragments.

#### 2.4.2. Correlation Analysis of OPLS-DA-Derived Features from Control and Infected Tomato Plants

**Figure 5:**
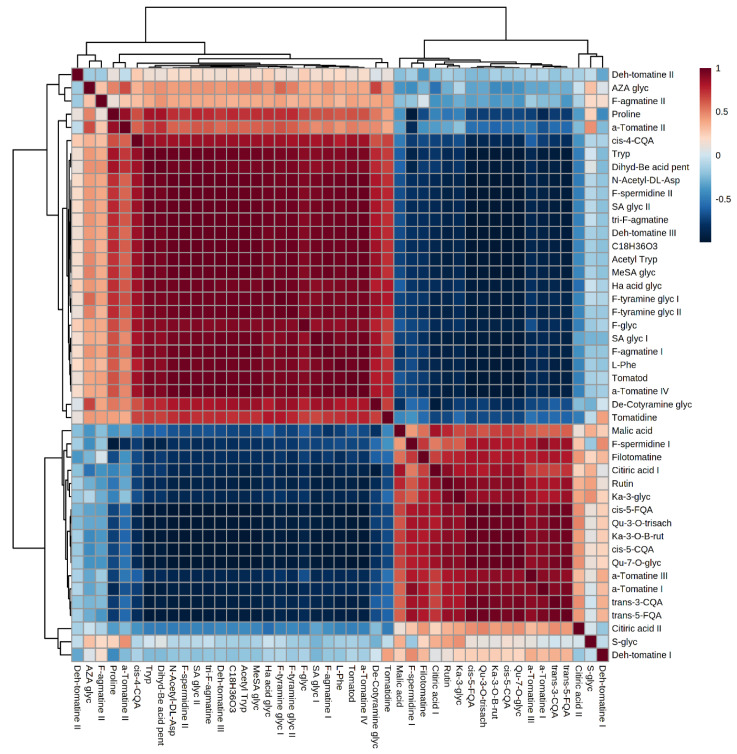
Correlation matrix among the changes (Δ) within/between extracts from leaves of non-treated (NT) day 8 tomato plants and *Phytophthora capsici* (PC)-treated plants at day 6 post-inoculation. Metabolite–metabolite correlations among identified molecules were obtained by deriving a Pearson correlation coefficient. Red indicates a positive correlation, and blue indicates a negative correlation. Abbreviations are explained in Table S1. Dendrograms are shown on the top and left of the correlation, indicating clustering of positive and negative correlations. The equivalent matrices for extracts from stems (site of inoculation) and roots are shown in Figures S16 and S17 respectively.

Following the annotation of significant features highlighted by OPLS-DA, Pearson correlation coefficient analysis was used to analyse the metabolite–metabolite correlation among identified molecules in NT day 8, and PC day 6 plants (Figure 5). Correlation measures the direction and strength of a linear relationship in bivariate data. Variables can be either positively/negatively correlated or not show any correlation. The former is a relationship where two variables move tandemly (one variable increases or decrease, and so does the other) and negative correlation is an inverse relationship between bivariate data (higher values of one variable are associated with lower values of the other). Thus, it allows identification of related metabolites in extracts from NT day 8 and PC day 6 plants. Fifty five significant metabolites were annotated in the different tissues (root, stem, and leaf) and metabolite–metabolite correlation of these significant biomarkers between NT day 8 and PC day 6 showed a unique profile in different samples (Figure 5).

**Figure 6:**
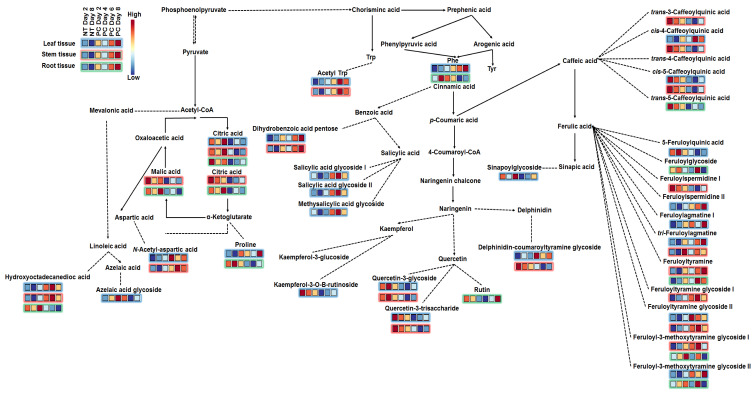
Metabolic changes in *Phytophthora capsici*-infected plant tissues (roots, stems, and leaves) over the time period of 2–8 d.p.i. Selected metabolites (VIP score ≥ 0.5) in infected plants were compared to those in control plants at the given time point. Blue boxes indicate down regulated metabolites/associated with the control, while red boxes indicate up regulated metabolites/associated with the treatment. Solid lines with an arrow indicate a single reaction, dotted lines with an arrow indicate multiple reactions, and dotted lines with no arrows indicated conjugation. NT = non-treated and PC = *P. capsici,* (stem-inoculated).

Notably, positive and negative correlation was observed between metabolites derived from the same biochemical pathway, but also between metabolites in entirely different pathways. For example in root tissue (Figure S17) the amino acids Phe and Pro have a strong positive correlation (*r* = 1). Furthermore, these amino acids have a positive correlation with dehydro-tomatine I and dihydro-benzoic acid pentose, but a negative correlation with acetyl Trp (Figure S17). In stem tissue, Pro and Trp have a positive correlation, but have a strong negative correlation with Phe and N-Acetyl-Asp (Figure S16). In addition, chlorogenic acids (*cis*-4-CQA and *trans*-3CQA) have a strong positive correlation. However, these molecules have a strong negative correlation with two other hydroxycinnamic acid (HCA) conjugates, (feruloyl-tyramine and feruloyl-agmatine II) (Figure S16). A similar trend was also observed in leaf tissue (Figure 6). Here, flavonoids, kaempferol-3-O-B-rutinoside and quercetin-3-O-trisacharide, are positively correlated (*r* = 1) but are negatively correlated with salicylic acid glycoside and Phe (Figure 6). These results are in accordance with the notion that plants fine-tune their immune response based on the perceived stimulus. Furthermore, the metabolomics data reflects a complex/dynamic feedback mechanism caused by modulation (inhibition/activation) of enzymes involved in the production of metabolites in the same pathway or in different pathways.

#### 2.4.3. Time-Course of Comparative Metabolite Reprogramming in *P. capsici*-Infected Tomato Plants

To further mine the data, and in order to derive greater biochemical insights into the underlying biochemistry of the host response to infection, PLS-DA was computed to investigate time-dependent metabolic reprogramming of infected plants. To determine the response of each feature to *P. capsici* infection, measurements of selected metabolites (VIP score ≥ 0.5) in infected plants were compared to those in control plants at the given time point, and observed a differential metabolic reprogramming (Figure 6). This resulted in pinpointing a total of 30 reprogrammed metabolites over a time period of 8 days. These belonged to the classes of flavonoids, fatty acids, amino acids, TCA intermediates, glycoalkaloids, and HCA derivatives, representing the core metabolome responsive to *P. capsici* infection. These discriminant molecular features of Figure 6 are in accordance with the ions depicted in the S-plots derived from the OPLS-DA models (Figure 4A and Figures S11A–S15A), and further explain the time-dependent clustering observed in the PCA (Figure 3A and Figures S6A–S10A) and HC analysis (Figure 3B and Figures S6B–S10B) models.

##### Differential Reprogramming in Primary Metabolism

The host response of the tomato plant to *P. capsici* treatment exhibited a contrasting metabolic reprogramming in the different tissues and in the regulated pathways (Figure 5 and Figure 6, Figures S16 and S17). Using the MS and MS/MS spectra, three amino acids (Pro, Phe, and Trp), two amino acid derivatives (N-acetyl-tryptophan and N-acetyl-aspartic acid), and two organic acids (citric acid I/II and malic acid), derived from the TCA cycle, were annotated (Figure 6). The two citric acids were found in all tissues and decreased with disease progression in root and stem tissue, whereas in leaf tissue, citric acid I increased with disease progression, and citric acid II decreased. In addition, the TCA cycle led to the production of fatty acids, and one oxygenated fatty acid (octadecanoic acid derivative) was annotated. C_18_H_36_O_3_ was found in all tissues (Figure 6). In both leaf and stems C_18_H_36_O_3_ was found to increase with disease progression, with maximum content on day 6 (Figure 6), whereas the opposite was observed in roots.

##### Differential Reprogramming of Phytohormones and Signalling Molecules

Azelaic acid (Aza)-glycoside was identified in both stem and leaf tissue on day 6 post-inoculation (Figure 6). However, the VIP score was 0.5 in both tissues, hence no time-trend was observed. One salicylic acid-glycoside (SAG) was found in stems, while in leaves two SAGs and methyl salicylic acid glycoside (MeSAG) were annotated. Furthermore, a time-trend could only be observed in leaf tissue, and all three molecule showed an increase during disease progression. SA is a well-documented phytohormone responsible for resistance induction following pathogen infection, and MeSA is the volatile derivative, able to move systemically and trigger distal responses [11,13].

##### Differential Reprogramming of Flavonoids and Hydroxycinnamic Acid Derivatives

The results revealed flavonoids as signatory biomarkers during *P. capsici* infection, and were shown to be reduced in infected plants (Figure 6). Flavonoids are phenolic molecules that play various functions in plants (e.g., signalling, antioxidant, biotic, and abiotic stress resistance) and their occurrence in plants during plant defences is geared toward protective and defence activities. HCA derivatives were annotated as discriminant biomarkers, and found to be conjugated to sugars, polyamines, and quinic acids (Figure 6). Furthermore, these molecules showed a contradicting content (decrease or increase) during pathogen infection.

##### Differential Reprogramming of Glycoalkaloids

**Figure 7:**
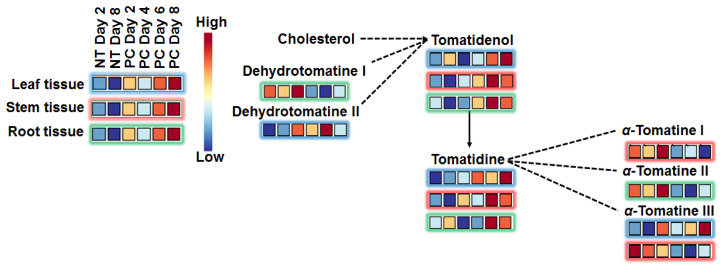
Metabolic changes in glycoalkaloid pathway in *Phytophthora capsici*-infected tomato tissues over the time-period of 2–8 d.p.i. Selected metabolites (VIP score ≥ 0.5) in infected plants were compared to those in control plants at the given time point. Blue boxes indicate down regulated metabolites/associated with the control, while red boxes indicate up regulated metabolites/associated with the treatment. Solid lines with an arrow indicate a single reaction, dotted lines with an arrow indicate multiple reactions, and dotted lines with no arrows indicate conjugation. NT = non-treated and PC = *P. capsici* (stem-inoculated).

Glycoalkaloids were identified among the annotated significant features/discriminatory biomarkers, and time-differential accumulation was observed (Figure 7). For example in roots, the content of these molecules was found to be high at different time points, or high on day 2, and then decreased thereafter. Similar trends were observed in both stem and leaf tissues.

## 3. Discussion

*P. capsici* is a hemibiotrophic pathogen with an initial biotrophic phase, followed by a switch to a necrotrophic lifestyle [1,2,3]. It is not known what triggers this switch (e.g., stress signals originating from the host). Following establishment of a successful biotrophic infection at the initial site of inoculation, localised defense responses are triggered, which may elicit further/subsequent systemic responses. While biotrophy is associated with SA-mediated plant defense, the switch to necrotrophy (depending on penetration into the endodermis and vasculature and movement along the xylem) leads to cell death, and production of ethylene and jasmonic acid, and derivatives [2,3,49]. Moreover, cell death of the roots and stems will limit or prevent transfer of water and solutes to the foliage. The observed wilting symptoms of the leaves may therefore be associated with abiotic stress responses, superimposed on the biotic stress responses. It is therefore conceivable that the differential perturbations to the metabolomes of the roots, stems, and leaves are the refection of different stimuli, and that the tissue-specific changes in the leaves might be due to a combination of biotic and abiotic stresses.

Plant primary metabolism plays an important role in plant–pathogen interactions. Previous studies have shown that primary metabolites are “energy-reservoirs” for plant defence, and serve as precursors for secondary metabolite biosynthesis [25,27,50]. Among the different primary metabolites known to be involved in plant–pathogen interactions, amino acids are the most well-documented, and have been shown to be significantly modulated [27,51]. In the context of defence, aromatic amino acids synthesised from chorismate through the shikimate pathway, are especially important (Figure 1). These amino acids serve as a link to secondary metabolism as a source of important precursors, with regard to phenolic compound biosynthesis and lignin accumulation [51,52,53]. Metabolite profiling in *Arabidopsis thaliana* inoculated with an avirulent strain of *Pseudomonas syringae* pv. tomato DC3000 (*hrp*- mutant) revealed an increase in Trp, Tyr, Lys, Val, and Leu content, and a decrease in Glu, whereas inoculation with the virulent strain showed an increase in Ileu, Thr, Ala, Phe, Tyr, and Gln accumulation [54]. In addition, amino acids were found to be significantly affected by *Rhizoctonia solani* infection on various rice lines [55], as well as in tomato basal resistance and priming against *Botrytis cinerea* and *Ps. syringae* [56].

Pro is known to accumulate in plants in response to water and salt stress, where it functions as an osmolyte and protein stabiliser [57]. However, in this study, control plants had a high(er) content of Pro than infected plants (Figure 6), thus suggesting that Pro could be playing a different role than that under abiotic stresses. In this instance, Pro is used as a precursor for the biosynthesis of other molecules (providing both carbon and nitrogen) or is metabolised [58]. Phe belongs to the aromatic amino acid group and serves as a precursor for the phenylpropanoid pathway. Trp and its derivative (acetyl Trp) are involved in biosynthesis of indoles such as auxin [32,51], serotonin, and its HCA amides [59], which are known to play various functions during plant defence responses. This pathway produces numerous metabolites known to play major roles in plant defence response [52,60,61]. N-acetyl-Asp was only found in leaves and showed an increase during pathogen progression. In this regard, amino acid conjugation to other molecules is well documented; these can conjugate to phytohormones, and this has been found to be the regulatory hub of plant defence response [58].

Organic acids are carbon providers for the biosynthesis of metabolites and are intermediates of the TCA cycle [62]. In this study, citric, and malic, acids were annotated as biomarkers. Interestingly, tricarboxylates, like citric acid and fumaric acid, were reported to induce defense priming against bacteria in *Arabidopsis thaliana* [63]. The TCA cycle is a central metabolic pathway for aerobic processes, and is responsible for a major portion of carbohydrate, fatty acid, and amino acid oxidation, and which produces energy and reducing power [64,65]. The involvement of fatty acids in plant defence responses is still poorly understood. However, the molecules could be related to lipid signalling or precursor synthesis for later lipid peroxyl radical production, or membrane destruction accompanied with plant cell death infection [66]. In this context, an oxygenated fatty acid (octadecanoic acid derivative) was annotated. C_18_H_36_O_3_ was found in all tissues (Figure 6), and increased with disease progression in both leaf and stems.

Plant defence responses are highly regulated by phytohormones and the related signalling molecules [67,68]. SA and its derivative MeSA (Figure 2A), are key phytohormones, especially relevant for local and systemic acquired resistance (SAR) [11,13]. Due to its volatility, MeSA deserves special attention, as it can diffuse through membranes, thus activating systemic responses [67,69,70]. Similarly, ET is readily diffusible in plant tissues, exerting effects at very low concentrations. The gaseous state of ET makes it difficult to quantify, however, quantification of the precursor, ACC (Figure 2B), has been used to study ethylene perturbation by microbes or environmental stresses [71,72,73,74,75]. Moreover, some studies suggest that ACC also regulates plant development and defence responses [76,77]. Upon infection, these phytohormones accumulate at the site of ingress and are transported to non-infected plant parts where they activate defence responses [68,70]. It is of interest that MeSA concentrations were the highest in the stems (the site of inoculation, and where initial biotrophic growth will occur), while those of ACC (associated with necrotrophic growth) were the highest in the leaves. Previous studies have demonstrated that plant infection leads to reprogramming of phytohormone homeostasis (decrease or increase phytohormones) [21,72,73]. Infection of *A. thaliana* plants by three fungal pathogens *Alternaria brassicicola*, *Colletotrichum higginsianum,* and *Botrytis cinerea* caused minor effects on MeSA and ACC levels [73]. Moreover, studies have shown the ability of fungal pathogens such as *Botrytis cinerea*, *Ustilago maydis*, *P. sojae,* and *Magnaporthe oryzae* to suppress phytohormone signalling by different mechanisms. These include conversion of phytohormones to inactive products or degradation of precursors [78]. 

Chemometric analyses revealed a significant accumulation of the phytohormones azelaic acid-glycoside, salicylate-glucoside, and methyl salicylic acid glucoside (Figure 6) as a differentiating characteristic of the defence responses of tomato plants to *P. capsici* infection. Phytohormones coordinate multiple physiological and biochemical processes, such as plant growth and development, gene regulation, and responses to abiotic and biotic stresses [4,79]. Aza is an emerging plant signalling molecule involved in SAR induction and regulation, and acts to confer resistance in both local and distal plant tissue [80]. The accumulation of various Aza derivatives including Aza-glycoside in lipopolysaccharide (LPS)-treated tobacco cell suspensions was reported [81], and the presence of Aza-glucosyl transferase(s), involved in Aza glycosylation, was proposed. The accumulation of Aza-glycoside in stem (site of infection) and leaf (distal) tissue, emphasises the link between local and distal signalling. Glycosylation of aglycones could serve both a regulatory and transport function. SA can be methylated to produce the mobile form MeSA, which can be transported to distal tissues, where it is demethylated to SA [13,81,82]. Studies have indicated that, after successful defence, the free active SA is glycosylated into inactive SAG, and the less abundant SA-derivative salicyloyl glucose ester (SAGE) by SA glucosyl transferases for storage [81,83].

Flavonoid compounds may accumulate in high quantities during pathogen infection [84]. Some metabolic studies have shown that tomato plants infected with *B. cinerea* had a higher content of flavonoids compared to non-infected plants [56]. In addition, in potato plants, accumulation of flavonoids was associated with resistance against late blight caused by *P. infestans* [85]. However, the results for *P. capsici* infected tomato showed a general decrease in flavonoid content during pathogen progression (Figure 6); an unexpected phenomenon that has not been well-described in the literature. This decrease in flavonoid content could be associated with defence response specificity (plants use similar pathways to defend against pathogens, albeit differently), re-channelling of HCA precursors for biosynthesis of other molecules such as phytoalexins or incorporation to the cell wall [2,61,84]. If not replenished, levels of flavonoids can also exhibit a decrease, if degraded by the pathogen, or chemically altered due to oxidative environments and antioxidant reactions.

Relatedly, the differential accumulation of HCA derivatives vs. flavonoids is an indication that the metabolic pools change with disease progression, as reported in a study of tomato cultivars responding to *Ralstonia solanacearum* infection [61]. Defence-related accumulation of HCA derivatives has been well-documented [61]. Ferulic-, caffeic-, *p*-coumaric-, and sinapic-acids are functional antimicrobial compounds, and precursors to the synthesis of both inducible and constitutive defence metabolites. They are also key in structural defences, as monolignol precursors of lignin, and by participating in cross-linking primary cell wall polysaccharides. Furthermore, HCA amides derivatives, such as 4-coumaroylagmatine and feruloylserotonin, annotated in this study, are also known in the context of cell wall strengthening, as well as antimicrobial compounds [86].

Glycoalkaloids occur naturally in tomato plants [66]; however, studies have shown that these molecules have antimicrobial properties against various plant pathogens, suggesting a major role in disease resistance [2,61]. Tomatidine is an interesting biomarker (Figure 7) because of the ability of bacteria and fungi to remove one or more sugar residues, thus rendering it less toxic. It is a well-known fact that microbes have evolved to suppress plant defence responses. Genes encoding glycoside hydrolases with potential activity against glycoalkaloids have been proposed in *P. infestans*, while evidence of deglycosylation has been reported [2].

## 4. Materials and Methods

### 4.1. Plant Growth Conditions and Treatment with P. capsici

A virulent strain of *Phytophthora capsici* (PRRI 20101) was obtained from the Plant Protection Institute, Agricultural Research Council (ARC), Pretoria, South Africa. Tomato (*Solanum lycopersicum* var. Moneymaker) seeds were germinated and grown in potted, washed, and autoclaved playpen sand under controlled conditions [87]. The greenhouse conditions were as follow: min temperature 15 °C and max temperature 28 °C, light/dark cycle of 12/12 h, and light intensity of 60 μmol/m^2^/s. Watering and fertiliser application were done on a weekly basis. The fertiliser consisted of: 650 mg/L CaNO_3_, 400 mg/L KNO_3_, 300 mg/L MgSO_4_, 90 mg/L mono-ammonium phosphate, 90 mg/L mono-potassium phosphate, 150 mg/L Soluptase, 20 mg/L Microples, and 40 µL/L Kep-P-Max, obtained from Shiman (Olifantsfontein, South Africa) [88]. The plants were grown for 8 weeks before infection. *P. capsici* was grown on potato dextrose agar in Petri dishes at 28 °C for 5 d. Small triangular pieces were cut from the 5 d old culture and used to inoculate oatmeal agar. Spores were harvested from 2 week old cultures by cold shocking at −20 °C for 20 min followed by sonication in a sonicating bath for 2 min and filtration through two layers of Miracloth. Zoospores were microscopically counted using a hemocytometer and adjusted to 1 × 10^6^/mL for inoculation with sterile water. The plants were inoculated by pipetting 500 µL of the spore solution onto a clean Ventti filter wrapped around a wounded stem. Stems were wounded by cutting a small section with a scalpel blade. Control plants were also wounded and wrapped with a Ventti filter; however, sterile water was used instead of the spore solution. The plants were kept in the growth room, and harvested every second day until the 8th day (PC 2, 4, 6, 8). Since there were no major differences observed in the control plants, control (non-treated) plants were harvested at day 2 and day 8 (NT 2 and NT 8). Three plants were harvested per condition, and each plant was treated as a biological replicate. Together with the three analytical replicates per biological replicate, this generated *n* = 9 as required for metabolomic analysis. Following harvesting, the plant tissue (roots, stems, and leaves) were snap-frozen in liquid nitrogen to quench metabolic activity and stored at −80 °C.

### 4.2. Targeted Metabolomics Analysis

Extraction of Amino Acids and Phytohormones, and UHPLC-QqQ-MS (Ultra High-Performance Liquid Chromatography Triple Quadrupole Mass Spectrometry) Analysis.

Following homogenisation of roots, stems, and leaf tissues, extraction was carried out as previously described [87]. The filtered 1.5 mL samples were analysed on a Nexera UHPLC (Shimadzu Corporation, Kyoto, Japan), fitted with a Restek Ultra AQ C18 column (100 mm × 2.1 mm × 3 µm) thermostatted at 40 °C, and coupled to a Shimadzu triple quad mass spectrometer (QqQ-MS) (Shimadzu Corporation, Tokyo, Japan). Chromatographic and mass spectrometry conditions were as previously described [87]. An internal standard of 1 ng/µL prednisolone (Pred) was included in the samples to monitor the instrument reliability and data acquisition reproducibility. LC-MS data acquisition was carried out in triplicate, and the results were expressed as mean values ± standard deviation (SD). Univariate analysis of variance (ANOVA) was performed as 2-tailed complete randomised blocks, and used to compare the non-infected vs. *P. capsici*-inoculated plants at different time points. ANOVA was followed by the Tukey post-hoc test, where differences between the means were considered significant at *p* < 0.05, indicated in graphs with an asterisk (*) or a dot (•). The summarised outputs are presented in Tables S2 and S3.

### 4.3. Non-Targeted Metabolomics Analysis

#### 4.3.1. Metabolite Extraction and Data Acquisition on an UHPLC-ESI-qTOF-MS (Ultra High-Performance Liquid Chromatography, Electrospray Ionisation, Quadrupole Time-of-Flight Mass Spectrometry)

Metabolite extraction and sample preparation were carried out as described [87]. The final filtered extracts were reconstituted to 300 µL (stems and leaves) and 250 µL (roots) in 50% methanol. A pooled biological quality control (PBQC) sample was prepared by pipetting and mixing aliquots of equal volumes from the samples. The samples were analysed in triplicate on an UHPLC system (Waters Acquity HSS T3 C18 column, 150 mm × 2.1 mm × 1.8 µm, thermostatted at 60 °C), coupled to high-definition MS (UHPLC-HD-MS), controlled and operated by MassLynx XS^TM^ software (Waters, Milford, MA, USA). Both LC and MS conditions were as previously described [87] with minor MS setting modifications as follows: desolvation temperature, 450 °C; source temperature, 120 °C; capillary voltage, 2.0 kV; sample cone, 35 V; extraction cone, 4.0 V; desolvation gas (Nitrogen) flow, 550 L/h; cone gas (Nitrogen) flow, 50 L/h; detector voltage, 1700 V; scan speed, 0.1 sec; and interscan time, 0.02 s. PBQCs were used to assess the reliability and reproducibility of the LC-MS system. The samples were randomised and PBQC samples analysed every 10 injections. The MS settings were set to perform unfragmented and five fragmenting experiments (MS^E^) simultaneously, by ramping in-source collision energy from 3 to 30 eV [53,89,90,91].

#### 4.3.2. Multivariate Data Analysis

The acquired UHPLC-qTOF-MS raw data were pre-processed using MarkerLynx XS™ software (Waters, Milford, MA, USA), and raw data from both negative and positive modes of electrospray ioninsation (ESI) were analysed. For multivariate modelling the data matrices were created using the following parameters: Rt range, 2.5–25 min; Rt difference, 0.2 min; mass to charge ratio (*m*/*z*) range, 100–2000; *m*/*z* difference, 0.05; mass tolerance, 0.5; intensity threshold count, 10; and noise level, 3. The pre-processing parameters were adjusted (if needed) depending on visual inspection of the chromatograms. The resultant data matrixes obtained from MassLynx XS were imported into SIMCA-P version 14.0 (Umetrics, Umeå, Sweden), and Pareto scaling was applied for multivariate statistical analysis. Principal component analysis (PCA), an unsupervised method, was used to summarise the information content in the datasets, thus providing “smaller indices” that could be easily visualised and analysed. Orthogonal-partial least square discriminant analysis (OPLS-DA), a supervised method, was used to extract maximum information of significant variables from the datasets. Method validation and variable selection were as described [87]. 

#### 4.3.3. Metabolite Annotation

Significant/discriminant features identified from the multivariate data analysis were annotated as described [87]. In short, a single ion chromatogram for each significant feature was extracted, its accurate mass determined, and its corresponding spectrum used to calculate an empirical formula to search in databases such as the Dictionary of Natural Products [92] and ChemSpider [93]. In addition, the spectra were compared with published data.

#### 4.3.4. Metabolite- Metabolite Correlation and Time-Dependent Reprogramming

Following annotation of discriminatory markers, the identified metabolites were extracted from the MarkerLynx XS™ generated matrixes and saved as .csv files. These were uploaded to MetaboAnalyst 4.0 [94] for further statistical analysis. The files comprised a list of experimental conditions, compound names, and intensities. MetaboAnalyst data processing performs checks on data integrity and missing values, and data filter and normalisation, prior to statistical analysis [95,96]. Correlation analysis was performed on non-treated (NT, day 8) and *P. capsici* (PC)-treated day 6 (based on OPLS-DA observations) samples to investigate the linear relationship between the annotated metabolites. Subsequently, partial least square-discriminant analysis (PLS-DA) (a supervised method) was applied to investigate time-dependent metabolic reprogramming in NT vs. PC-treated plants.

## 5. Conclusions

LC-MS targeted analysis showed a time-dependent regulation of aromatic amino acids as part of the host response of tomato plants in response to infection by *P. capsici*. The Phe and Trp content increased upon infection, while Tyr increased up to day 4 and gradually decreased thereafter. These findings show the importance of primary metabolites, mainly aromatic amino acids, which are involved in the biosynthesis of phenylpropanoids, flavonoids, and indoles. The increases indicate the demand for phenolic precursors, in comparison to the Tyr decrease, which suggests that the consumption is higher than synthesis. Furthermore, untargeted LC-MS metabolomics analysis showed time-dependent metabolic changes in the non-treated vs. *P. capsici*-infected plant tissues. The annotated metabolites included phenylpropanoids, benzoic acids, glycoalkaloids, flavonoids, amino acids, and TCA cycle organic acids, as well as oxygenated octadecanoic acids. Metabolite–metabolite correlations showed that there was a dynamic regulation of the programmed metabolites, as evidenced by positive and negative correlation among the significant biomarkers. Lastly, tissue-specific reprogramming was also observed, which demonstrated that various plant tissues (i.e., roots, stems, and leaves) undergo differential metabolomic changes in response to infection. Here, intrinsic tissue-specific profiles would also be influenced by localised infection in the stem, the biotrophy to necrotrophy switch, and the balance between generated signalling molecules, able to trigger systemic responses, as well as possible abiotic stress responses linked to wilting. Regardless, the perturbations of the metabolomes can be interpreted as due to the activation of chemical defences against the pathogen, in an attempt to ward off the infection or limit the cellular damage caused by the infection. The relative importance of the identified metabolic pathways cannot be judged with the available data; however, the identified biomarkers could pave the way for further studies in the tomato response to *P. capsici* infection.

## Data Availability

The study design information, LC-MS raw data, analyses and data processing information, and the meta-data were deposited to the EMBL-EBI metabolomics repository MetaboLights50, https://www.ebi.ac.uk/metabolights/MTBLS1176.

## Supplementary Materials

The Supplementary Materials are available online at https://www.mdpi.com/2218-1989/10/11/466/s1.
