# Supplementary material for: Concurrent Metabolic Profiling and Quantification of Aromatic Amino Acids and Phytohormones in Solanum lycopersicum Plants Responding to Phytophthora capsici
Source: Metabolites. 2020 Nov 16;10(11):466. doi: 10.3390/metabo10110466 (PMC7696014; doi:10.3390/metabo10110466)
Supplement: Supplementary file 1 [file metabolites-10-00466-s001.docx]

*Research article – Supplemenatry data*

**Concurrent metabolic profiling and quantification of aromatic amino acids and phytohormones in *Solanum lycopersicum* plants responding to *Phytophthora capsici*****.**

**Msizi I. Mhlongo^1^, Lizelle A. Piater^1^, Paul A. Steenkamp^1^ Nico Labuschagne^2^ and Ian A. Dubery^1^***

^1^Department of Biochemistry, University of Johannesburg, P.O. Box 524, Auckland Park, Johannesburg, South Africa; ^2^Department of Plant and Soil Sciences, University of Pretoria, Private Bag X20, Hatfield, Pretoria, 0028, South Africa. * Correspondence: E-mail: idubery@uj.ac.za; Tel.: +27-11-559-2401.

__________________________________________________________________________________________


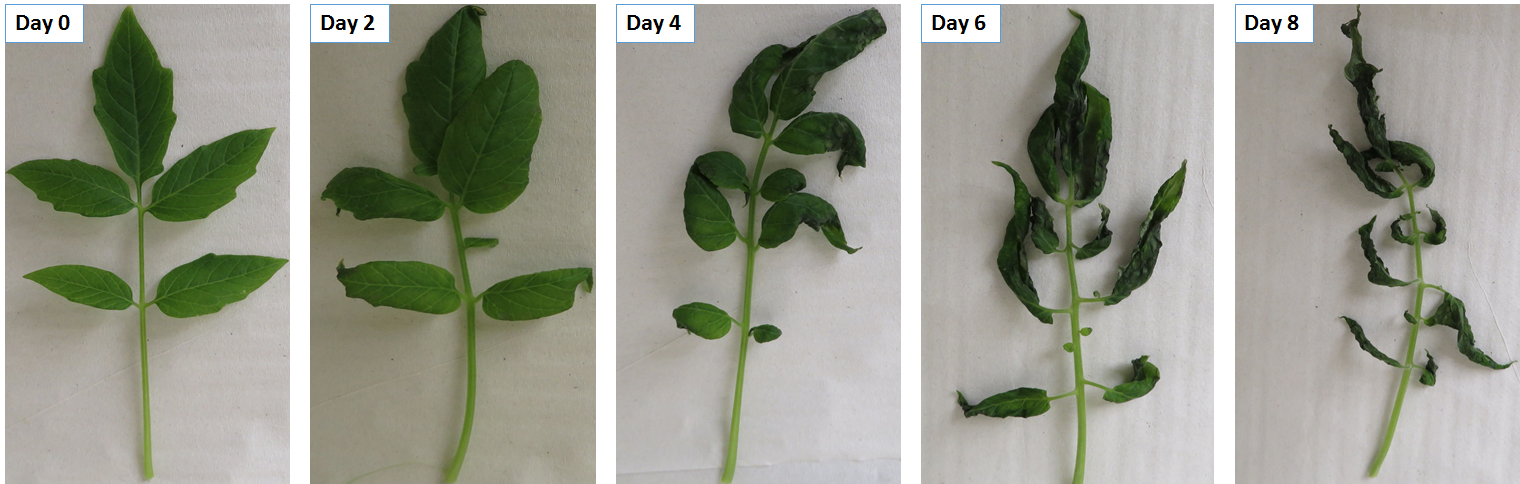


**Fig. S1: *S*ymptom development** i**n tomato leaves harvested at different time points following inoculation with *Phytophthora capsici* zoospores**. Leaf wilting started on day 2 and progressed over time, and at day 8 the tomato leaves were completely wilted.

**
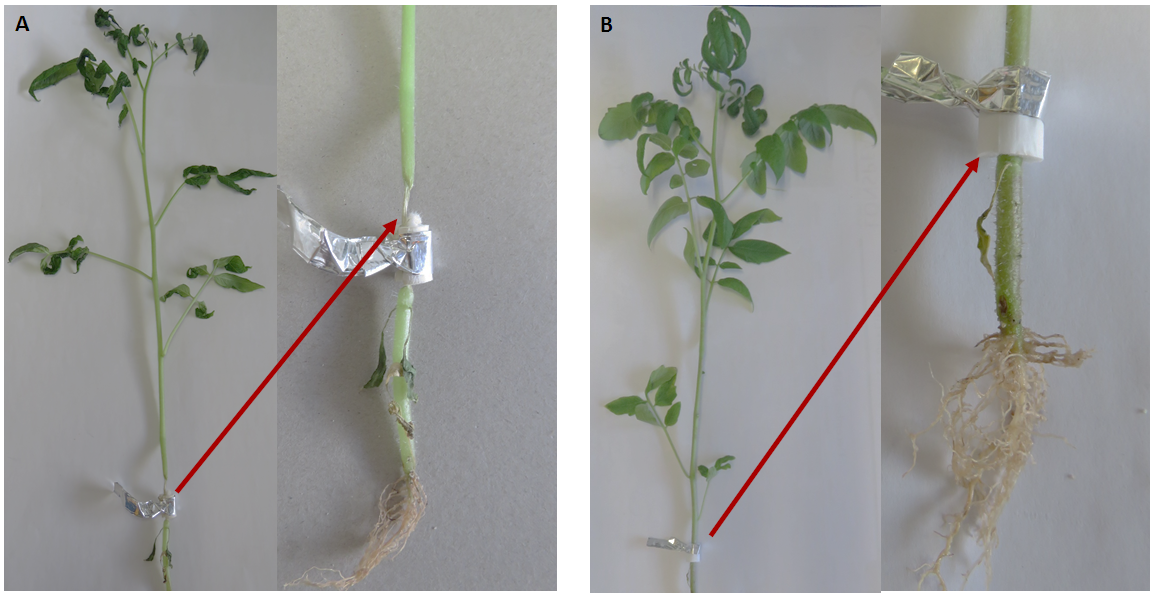
**

**Figure S2: Eight days post-infection comparison between *P. capsici* -infected and control tomato plants.**

**(A):** Zoospore inoculated plant with arrow indicating infection and necrosis at the inoculation site and

**(B):** Control plant with arrow pointing to a Ventti filter wrapped around the inoculation site. (Section 4.1).


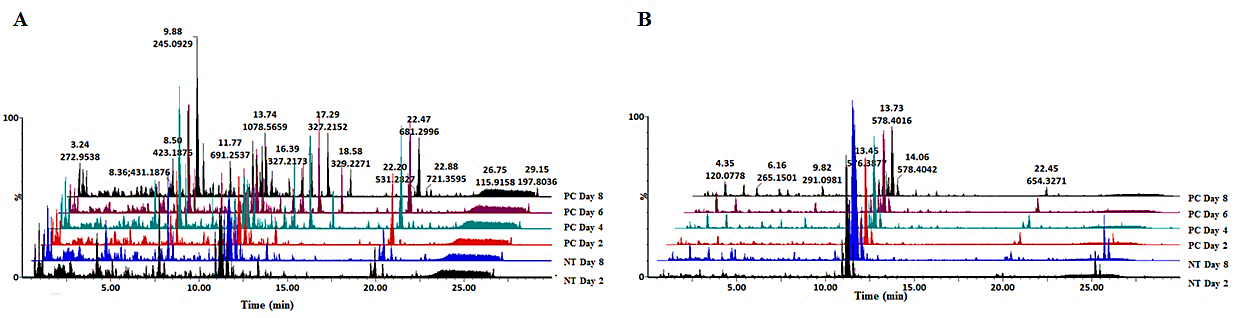


**Figure S3: Representative BPI MS chromatograms of extracts from tomato plants infected with *Phytophthora capsici;* leaf tissue.** Base peak mass chromatograms displaying comparative chromatographic differences in different time points: non-treated (NT, days 2 and 8) and *P. capsici* (PC, days 2, 4, 6 and 8) infected. Visual inspection of the chromatograms evidently shows differential peak populations, for instance in the **4-20 min** chromatographic region. **(A):** ESI negative mode and **(B):** ESI positive mode.


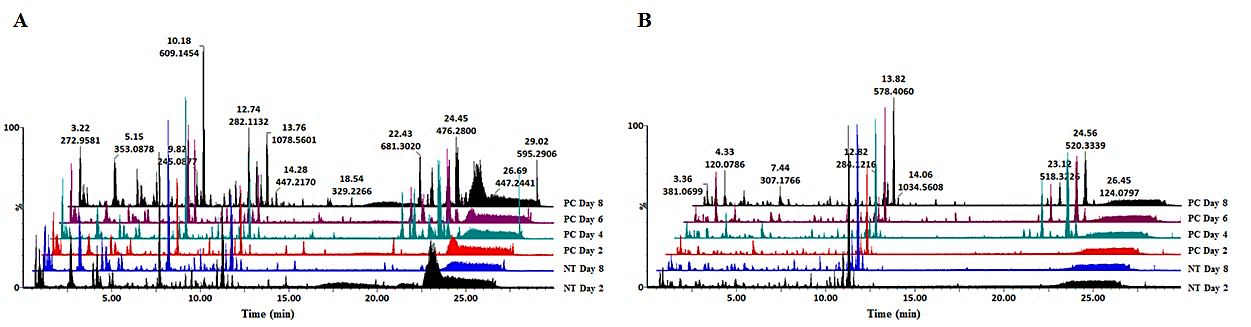


**Figure S4: Representative BPI MS chromatograms of extracts from tomato plants infected with *Phytophthora capsici;* stem tissue.** Base peak mass chromatograms displaying comparative chromatographic differences in different time points: (i) non-treated (NT, days 2 and 8) and (ii) *P. capsici* (PC, days 2, 4, 6 and 8) infected. Visual inspection of the chromatograms evidently shows differential peak populations, for instance in the **4-20 min** chromatographic region. **(A):** ESI negative mode and **(B):** ESI positive mode.


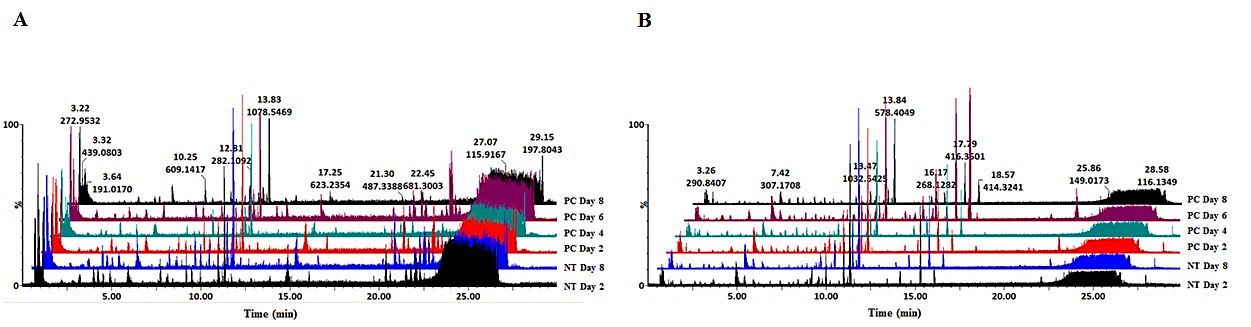


**Figure S5: Representative BPI MS chromatograms of extracts from tomato plants infected with *Phytophthora capsici;* root tissue.** Base peak mass chromatograms displaying comparative chromatographic differences in different time points: (i) non-treated (NT, days 2 and 8) and (ii) *P. capsici* (PC, days 2, 4, 6 and 8) infected. Visual inspection of the chromatograms evidently shows differential peak populations, for instance in the **4-20 min** chromatographic region. **(A):** ESI negative mode and **(B):** ESI positive mode.


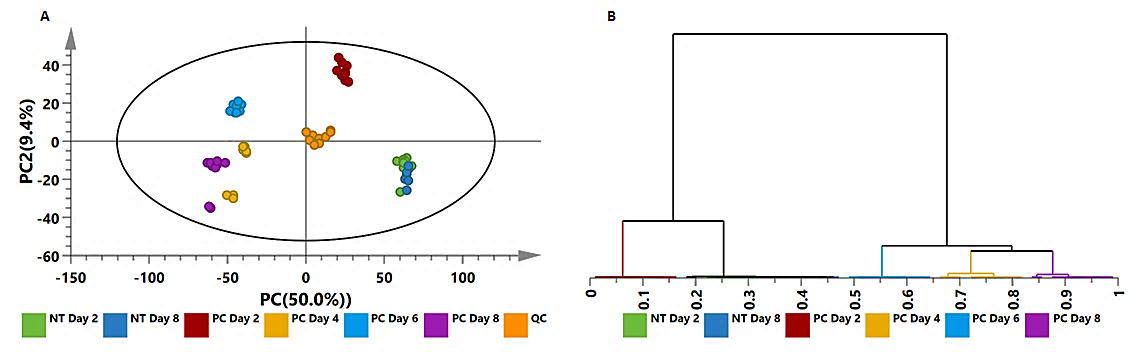


**Figure S6: Unsupervised statistical analysis of extracts from tomato plants infected with *Phytophthora capsici;* leaf data acquired in ESI^+^ mode.** **(A):** A PCA scores scatter plot of all the samples, including the QC samples, colored according to time points. The PCA model presented here was a 7-component model, with R^2^ of 0.697 and Q^2^ of 0.645. **(B):** The HCA dendrogram corresponding to **(A)**. Unsupervised statistical analysis is used to generate subgrouping of samples based on similar observations in **(A)** while the HCA dendrogram shows the hierarchical relationship between samples **(B)**.


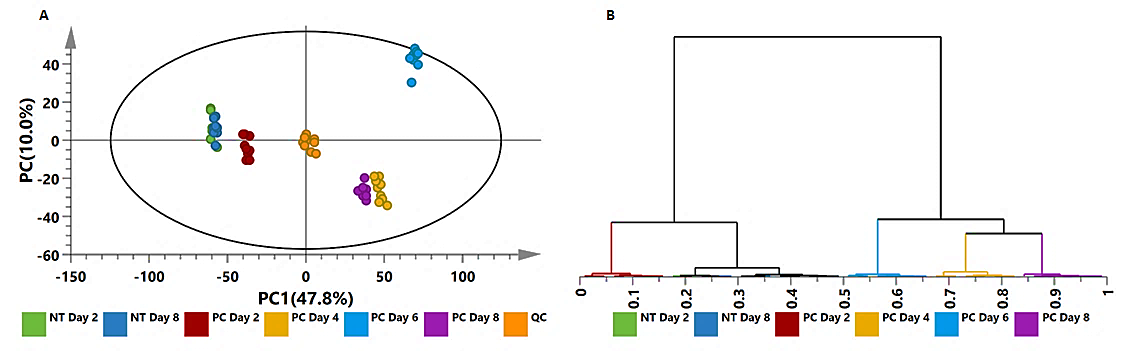


**Figure S7: Unsupervised statistical analysis of extracts from tomato plants infected with *Phytophthora capsici;* stem data acquired in ESI^-^ mode.** **(A):** A PCA scores scatter plot of all the samples, including the QC samples, colored according to time points. The PCA model presented here was a 7-component model, with R^2^ of 0.804 and Q^2^ of 0.752. **(B):** The HCA dendrogram corresponding to **(A)**. Unsupervised statistical analysis is used to generate subgrouping of samples based on similar observations in **(A)** while the HCA dendrogram shows the hierarchical relationship between samples **(B)**.


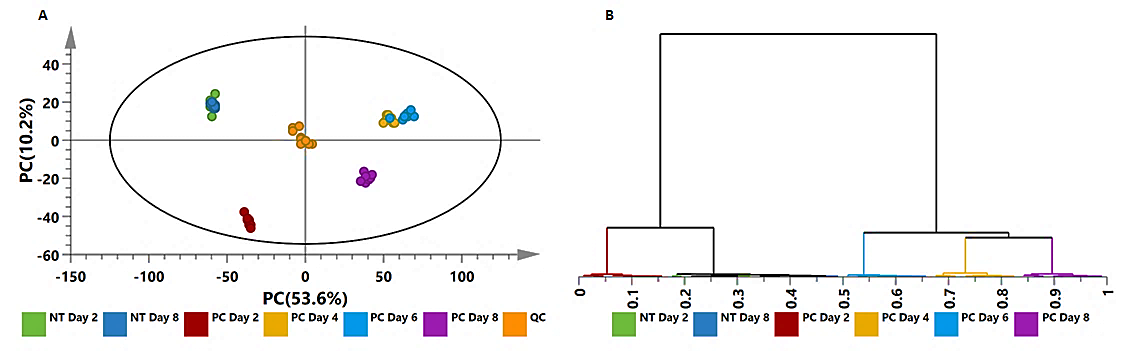


**Figure S8: Unsupervised statistical analysis of extracts from tomato plants infected with *Phytophthora capsici;* stem data acquired in ESI^+^ mode.** **(A):** A PCA scores scatter plot of all the samples, including the QC samples, colored according to time points. The PCA model presented here was a 7-component model, with R^2^ of 0.835 and Q^2^ of 0.783. **(B):** The HCA dendrogram corresponding to **(A)**. Unsupervised statistical analysis is used to generate subgrouping of samples based on similar observations in **(A)** while the HCA dendrogram shows the hierarchical relationship between samples **(B)**.


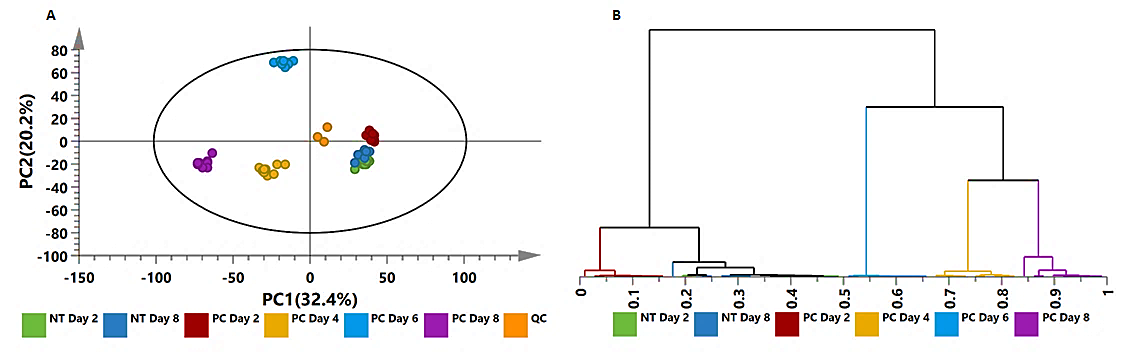


**Figure S9:** **Unsupervised statistical analysis of extracts from tomato plants infected with *Phytophthora capsici;* root data acquired in ESI^-^ mode.** **(A):** A PCA scores scatter plot of all the samples, including the QC samples, colored according to time points. The PCA model presented here was a 7-component model, with R^2^ of 0.759 and Q^2^ of 0.640. **(B):** The HCA dendrogram corresponding to **(A)**. Unsupervised statistical analysis is used to generate subgrouping of samples based on similar observations in **(A)** while the HCA dendrogram shows the hierarchical relationship between samples **(B)**.


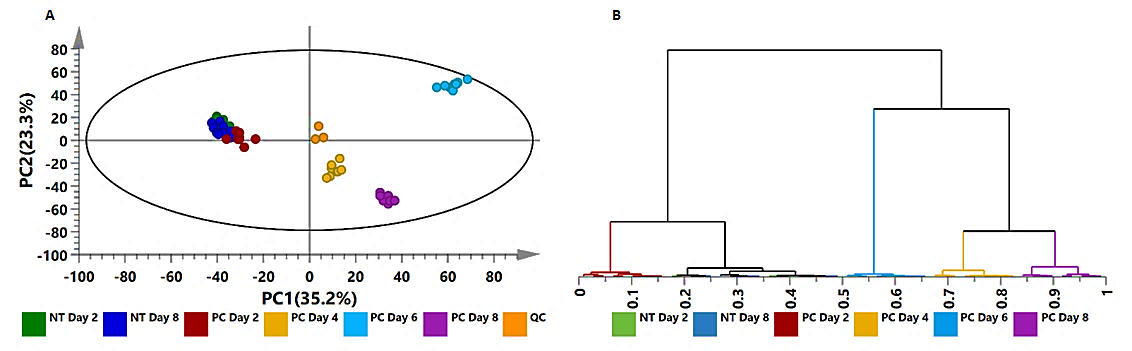


**Figure S10: Unsupervised statistical analysis of extracts from tomato plants infected with *Phytophthora capsici;* root data acquired in ESI^+^ mode.** **(A):** A PCA scores scatter plot of all the samples, including the QC samples, colored according to time points. The PCA model presented here was a 7-component model, with R^2^ of 0.788 and Q^2^ of 0.692. **(B):** The HCA dendrogram corresponding to **(A)**. Unsupervised statistical analysis is used to generate subgrouping of samples based on similar observations in **(A)** while the HCA dendrogram shows the hierarchical relationship between samples **(B)**.


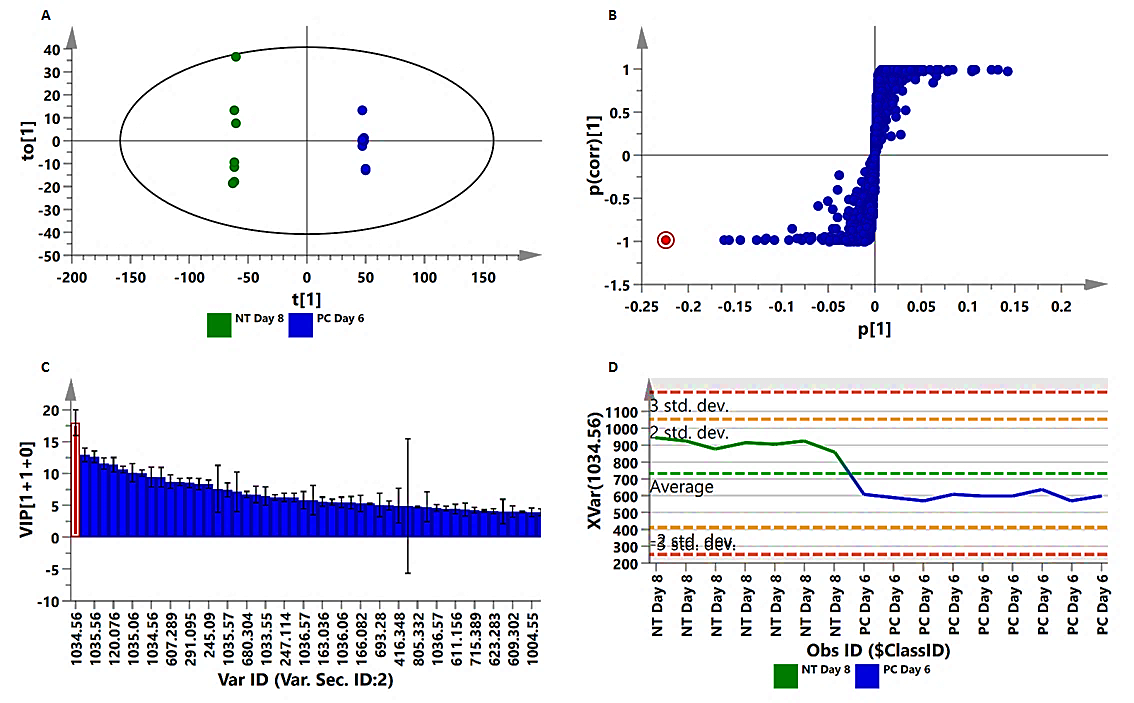


**Figure S11: OPLS-DA modeling and variable/feature selection of extracts from tomato plants infected with *Phytophthora capsici;*  leaf data acquired on ESI^+^ mode.** (**A**): A typical **OPLS-DA score plot** separating non-treated (NT) day 8 plants *vs.* *P. capsici* (PC)-treated day 6 plants (1 + 1 + 0 components, R^2^X = 0.741, Q^2^ = 0.998, CV-ANOVA *p*-value = 7.96 × 10^−15^). (**B**): An OPLS-DA **loadings S-plot** for the same model in (**A**); only variables with the correlation [*(p(corr)*] ≥ |0.6| and covariance *(p1)* ≥ |0.5| were chosen as discriminating variables and identified using the *m/z* to generate elemental composition. (**C**): A **variable importance for the projection (VIP) plot** for the same model, pointing mathematically to the importance of each variable in contributing to group separation in the OPLS-DA model. (**D**): A typical **variable trend plot** (of the selected variable in VIP and S-plots), displaying the changes of the selected variables across the samples (NT day 8 *vs.* PC day 6). This shows that the selected features significantly discriminate the treated from the control samples.


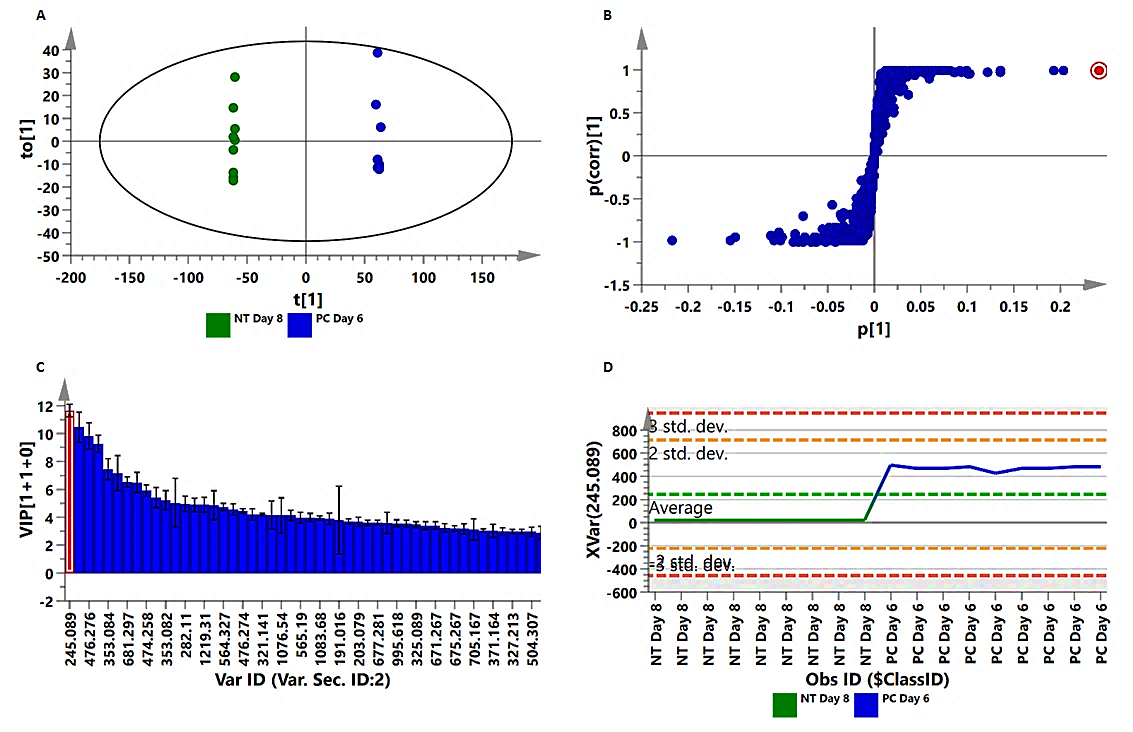


**Figure S12: OPLS-DA modeling and variable/feature selection of extracts from tomato plants infected with *Phytophthora capsici;* stem data acquired on ESI^-^ mode.** (**A**): A typical **OPLS-DA score plot** separating non-treated (NT) day 8 plants *vs.* *P. capsici* (PC)-treated day 6 plants (1 + 1 + 0 components, R^2^X = 0.817, Q^2^ = 0.999, CV-ANOVA *p*-value = 3.19 × 10^−20^). (**B**): An OPLS-DA **loadings S-plot** for the same model in (**A**); only variables with the correlation [*(p(corr)*] ≥ |0.6| and covariance *(p1)* ≥ |0.5| were chosen as discriminating variables and identified using the *m/z* to generate elemental composition. (**C**): A **variable importance for the projection (VIP) plot** for the same model, pointing mathematically to the importance of each variable in contributing to group separation in the OPLS-DA model. (**D**): A typical **variable trend plot** (of the selected variable in VIP and S-plots), displaying the changes of the selected variables across the samples (NT day 8 *vs.* PC day 6). This shows that the selected features significantly discriminate the treated from the control samples.


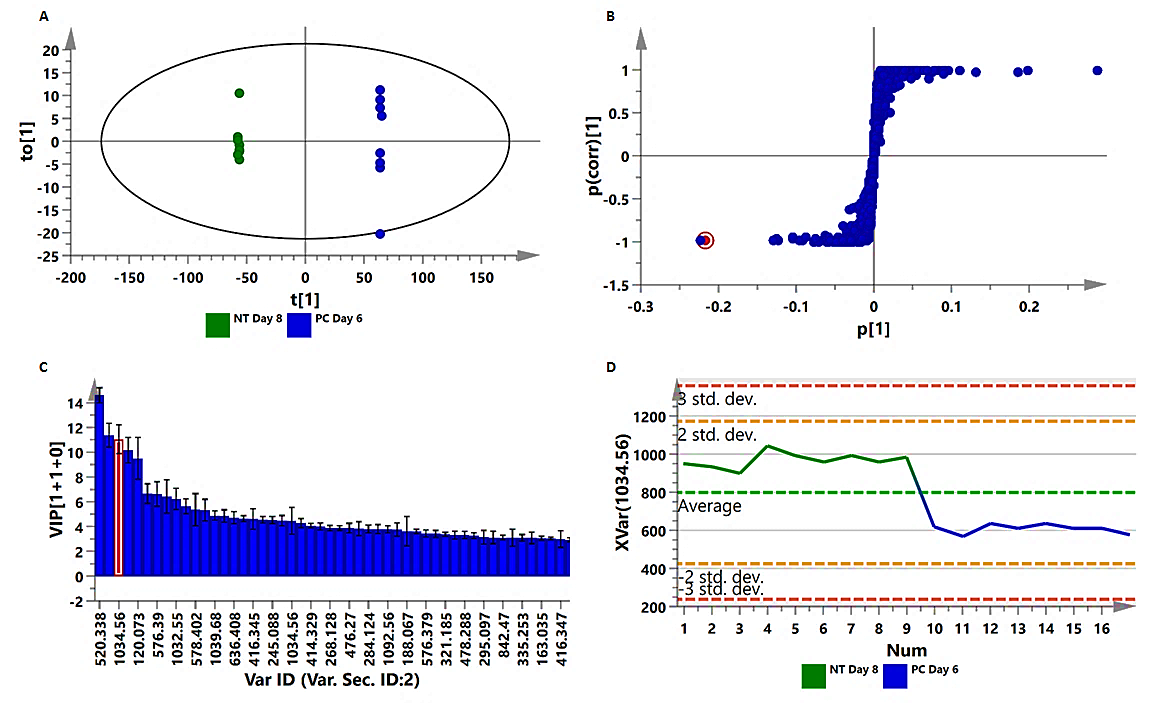


**Figure S13: OPLS-DA modeling and variable/feature selection of extracts from tomato plants infected with *Phytophthora capsici;* stem data acquired on ESI^+^ mode.** (**A**): A typical **OPLS-DA score plot** separating non-treated (NT) day 8 plants *vs.* *P. capsici* (PC)-treated day 6 plants (1 + 1 + 0 components, R^2^X = 0.855, Q^2^ = 0.995, CV-ANOVA *p*-value = 3.9 × 10^−18^). (**B**): An OPLS-DA **loadings S-plot** for the same model in (**A**); only variables with the correlation [*(p(corr)*] ≥ |0.6| and covariance *(p1)* ≥ |0.5| were chosen as discriminating variables and identified using the *m/z* to generate an elemental composition. (**C**): A **variable importance for the projection (VIP) plot** for the same model, pointing mathematically to the importance of each variable in contributing to group separation in the OPLS-DA model. (**D**): A typical **variable trend plot** (of the selected variable in VIP and S-plots), displaying the changes of the selected variables across the samples (NT day 8 *vs.* PC day 6). This shows that the selected features significantly discriminate the treated from the control samples.


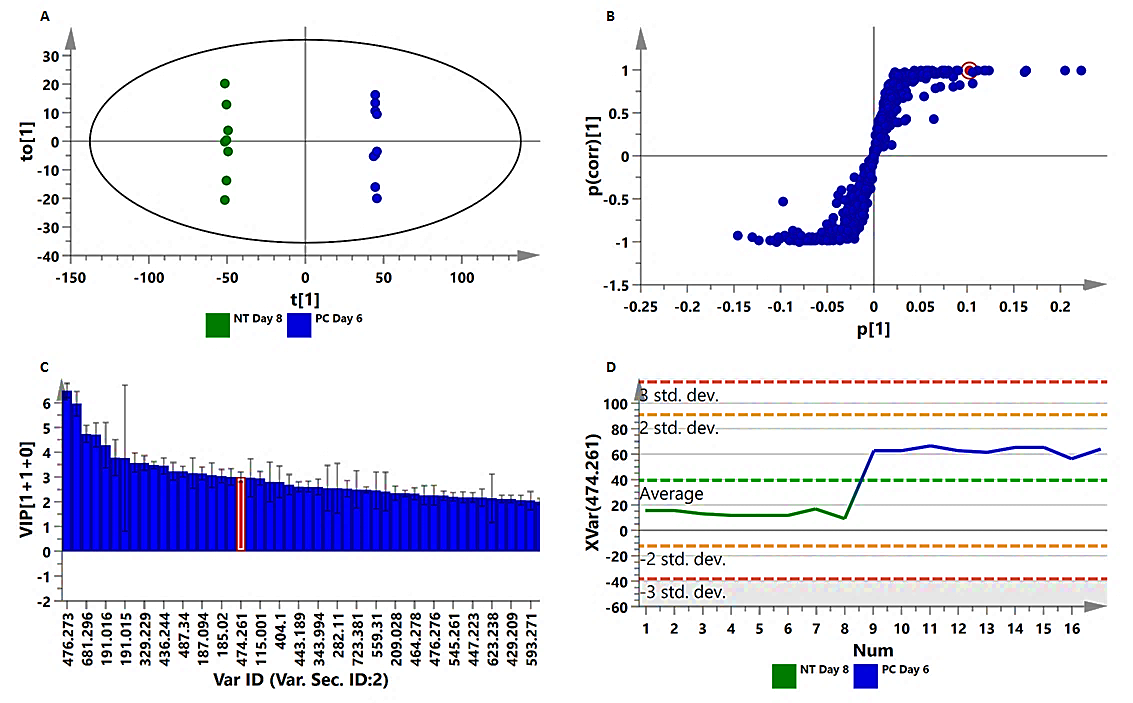


**Figure S14: OPLS-DA modeling and variable/feature selection of extracts from tomato plants infected with *Phytophthora capsici;* root data acquired on ESI^-^ mode.** (**A**): A typical **OPLS-DA score plot** separating non-treated (NT) day 8 plants *vs.* *P. capsici* (PC)-treated day 6 plants (1 + 1 + 0 components, R^2^X = 0.677, Q^2^ = 0.998, CV-ANOVA *p*-value = 9.48 × 10^−16^). (**B**): An OPLS-DA **loadings S-plot** for the same model in (**A**); only variables with the correlation [*(p(corr)*] ≥ |0.6| and covariance *(p1)* ≥ |0.5| were chosen as discriminating variables and identified using the *m/z* to generate an elemental composition. (**C**): A **variable importance for the projection (VIP) plot** for the same model, pointing mathematically to the importance of each variable in contributing to group separation in the OPLS-DA model. (**D**): A typical **variable trend plot** (of the selected variable in VIP and S-plots), displaying the changes of the selected variables across the samples (NT day 8 *vs.* PC day 6). This shows that the selected features significantly discriminate the treated from the control samples.


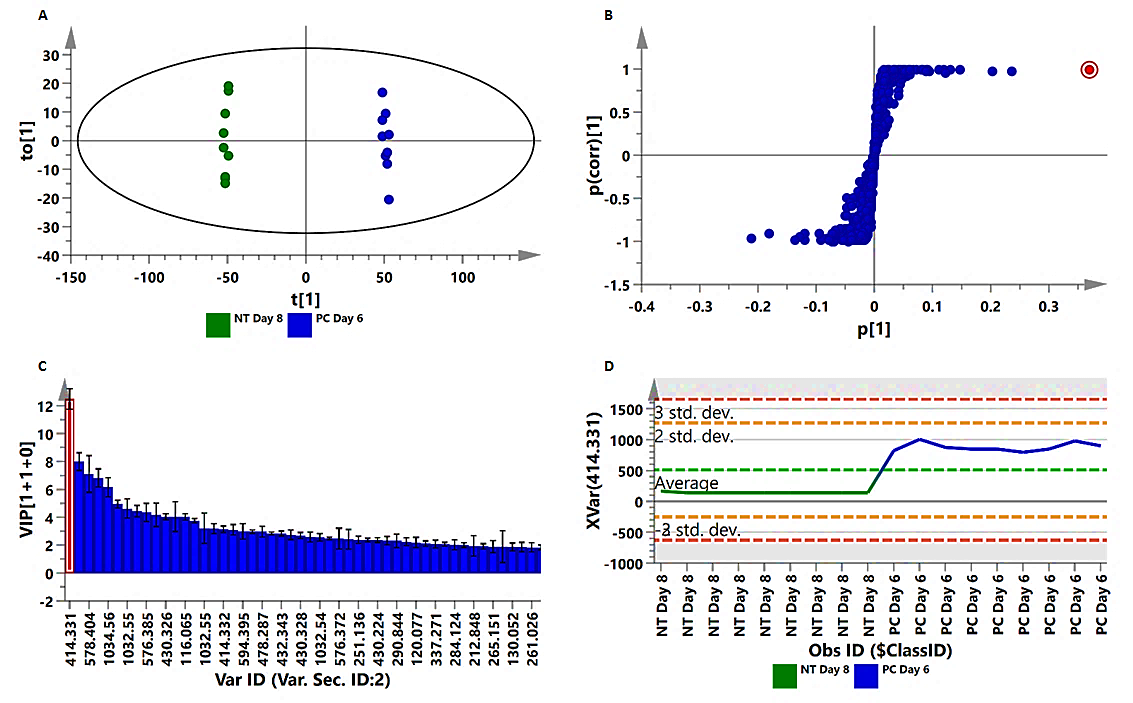


**Figure S15: OPLS-DA modeling and variable/feature selection of extracts from tomato plants infected with *Phytophthora capsici;* root data acquired on ESI^+^ mode.** (**A**): A typical **OPLS-DA score plot** separating non-treated (NT) day 8 plants *vs.* *P. capsici* (PC)-treated day 6 plants (1 + 1 + 0 components, R^2^X = 0.742, Q^2^ = 0.996, CV-ANOVA *p*-value = 1.08 × 10^−15^). (**B**): An OPLS-DA **loadings S-plot** for the same model in (**A**); only variables with the correlation [*(p(corr)*] ≥ |0.6| and covariance *(p1)* ≥ |0.5| were chosen as discriminating variables and identified using the *m/z* to generate an elemental composition. (**C**): A **variable importance for the projection (VIP) plot** for the same model, pointing mathematically to the importance of each variable in contributing to group separation in the OPLS-DA model. (**D**): A typical **variable trend plot** (of the selected variable in VIP and S-plots), displaying the changes of the selected variables across the samples (NT day 8 *vs.* PC day 6). This shows that the selected features significantly discriminate the treated from the control samples.

**Table S1: Summary of annotated (MSI-level 2) metabolites that contributed to the discriminating variability in the altered metabolomes of root -, stem - and leaf tissue of tomato plants infected with *Phytophthora capsici* (as described by chemometric models).** These discriminating metabolites were identified based on OPLS-DA S-plots, with a rigorous statistical validation (as explained in the text – Figure 4). These reported metabolites had VIP scores ˃ 1.0.

| **No.** | **Rt**  **(min)** | **Ionization** | ***m/z*** | **Compound name** | **Abbreviation** | **Chemical formula** | **Fragments**  **(*m/z*)** |
| --- | --- | --- | --- | --- | --- | --- | --- |
| **1** | 0.87 | [M-H]- | 191.018 | Citric acid I | C-acid I | C6H8O7 | 173, 115, 111 |
| **2** | 0.87 | [M+H]+ | 116.063 | L-Proline | L-Pro | C5H9NO2 | 70 |
| **3** | 0.92 | [M-H]- | 191.017 | Citric acid II | C-acid II | C6H8O7 | 173, 111 |
| **4** | 0.95 | [M-H]- | 133.007 | Malic acid | M-Acid | C4H6O5 | 114, 89, 72 |
| **5** | 1.85 | [M+H]+ | 166.082 | L-Phenylalanine | L-Phe | C9H11NO2 | 120, 103, 91, 77 |
| **6** | 2.19 | [M+H]+ | 220.117 | *N'*, *N'',N'''*-Triferuloylagmatine | tri-F-agmatine | C35H38N4O9 | 660 |
| **7** | 2.62 | [M+H]+ | 176.105 | *N*-Acetyl-aspartic acid | N-Acetyl-Asp | C6H9NO5 | 115 |
| **8** | 2.72 | [M-H]- | 353.084 | 3-Caffeoylquinic acid | 3-CQA | C16H18O9 | 191, 179, 135 |
| **9** | 2.92 | [M-H]- | 203.077 | Tryptophan | Trp | C11H12N2O2 | 142, 116 |
| **10** | 3.16 | [M-H]- | 285.058 | Dihydroxybenzoic acid pentose | diHydro-Be acid pent | C12H14O8 | 153 |
| **12** | 3.96 | [M-H]- | 343.186 | Homovanillic acid glycoside | H-acid glyc | C15H20O9 | 181 |
| **13** | 4.22 | [M-H]- | 353.083 | 5-Caffeoylquinic acid | 5-CQA | C16H18O9 | 191, 179, 173, 135 |
| **14** | 4.35 | [M-H]- | 367.099 | 4-Feruloylquinic acid | 4-FQA | C17H20O9 | 193 |
| **15** | 4.39 | [M+H]+ | 323.154 | *N*-Feruloylspermidine I | F-spe I | C17H27N3O3 | 321, 177, 145, 117 |
| **16** | 4.4 | [M-H]- | 353.082 | 4-Caffeoylquinic acid | 4-CQA | C17H20O9 | 191, 179, 173, 135 |
| **17** | 4.9 | [M-H]- | 355.098 | Feruloylglycoside I | F-glyco I | C16H20O9 | 193 |
| **18** | 4.94 | [M+H]+ | 307.17 | *N*-Feruloylagmatine I | F-agm I | C15H22N4O3 | 177, 145, 114 |
| **19** | 5.06 | [M-H]- | 385.107 | Sinapoylglycoside II | S-glyc I | C17H20O10 | 223 |
| **20** | 5.27 | [M+H]+ | 337.184 | *N*-Feruloylagmatine II | F-agm II | C15H22N4O3 | 177, 145, 114 |
| **21** | 5.42 | [M+H]+ | 322.187 | *N*-Feruloylspermidine II | F-spe II | C17H27N3O3 | 321, 177, 145, 117 |
| **22** | 6.21 | [M-H]- | 367.099 | 5-Feruloylquinic acid | 5-FQA | C17H20O9 | 191 |
| **23** | 7 | [M-H]- | 741.189 | Quercetin-3-O-trisacharide | Qu-3-O-trisach | C31H36O21 | 300 |
| **24** | 7.34 | [M-H]- | 245.089 | Acetyl tryptophan | Acetyl Trp | C13H14O3 | 203 |
| **25** | 7.7 | [M-H]- | 609.145 | Rutin | Rutin | C27H30O16 | 300 |
| **26** | 8.02 | [M-H]- | 463.083 | Quercetin 7-O-glucoside | Qu-7-O-gluc | C15H20O12 | 300 |
| **27** | 8.63 | [M-H]- | 593.149 | Kaempferol-3-O-B-rutinoside | Ka-3-O-B-rut | C27H30O15 | 285 |
| **28** | 9.48 | [M-H]- | 349.094 | Azelaic acid-glycoside | Aza-glyc | C15H26O9 | 187 |
| **29** | 9.97 | [M+H]+ | 1032.54 | Dehydrotomatine | De-tomatine | C50H81NO21 | 588, 576, 414 |
| **30** | 10.07 | [M+H]+ | 738.443 | Alpha tomatine I | A-tom I | C50H83NO21 | 738, 578, 416 |
| **31** | 10.26 | [M-H]- | 745.27 | Delphinidin-coumaroyltyramine glycoside | De-Cotyr glyc | C38H36NO14 | 282, 162, 119 |
| **32** | 10.7 | [M-H]- | 312.12 | Feruloyltyramine I | F-tyr | C18H19NO4 | 178, 134 |
| **33** | 10.73 | [M+H]+ | 1032.251 | Dehydrotomatine I | De-tomatine I | C9H81NO21 | 576, 414 |
| **34** | 10.97 | [M+H]+ | 1032.55 | Dehydrotomatine II | De-tomatine II | C50H81NO21 | 576, 414 |
| **35** | 11.1 | [M+H]+ | 344.146 | Alpha tomatine II | A-tom II | C50H83NO21 | 578, 416 |
| **36** | 11.11 | [M+H]+ | 344.147 | Filotomatine | Filo | C50H83NO21 | 578, 416, 207 |
| **37** | 11.13 | [M-H]- | 299.183 | Salicylic acid glycoside I | SA-glyc I | C13H16O8 | 137 |
| **38** | 11.2 | [M+H]+ | 1092.56 | Alpha tomatine III | A-tom III | C50H83NO21 | 578, 416, 295 |
| **39** | 11.56 | [M+H]+ | 1034.56 | Alpha-tomatine IV | A-tom IV | C50H83NO21 | 578, 416, 416, 295 |
| **40** | 11.66 | [M+H]+ | 1004.55 | Alpha tomatine V | A-tom V | C50H83NO21 | 578, 416, 295 |
| **41** | 11.8 | [M-H]- | 447.219 | Kaempferol-3-glucoside | Ka-3-O-gluc | C21H20O11 | 285 |
| **42** | 12.06 | [M-H]- | 299.182 | Salicylic acid glycoside II | SA-glyc II | C13H16O8 | 137 |
| **43** | 13.41 | [M+H]+ | 414.333 | Tomatidenol | Tomato I | C27H43NO2 | 273, 255, 161 |
| **44** | 13.42 | [M-H]- | 313.197 | Methyl salicylate glycoside | MeSA-glyc | C14H18O8 | 151 |
| **45** | 14.14 | [M+H]+ | 412.318 | Tomatid-4-en-3-one | Toma-one | C27H43NO2 | 325, 271, 161 |
| **46** | 14.75 | [M+H]+ | 414.334 | Tomatidenol | Tomato II | C27H43NO2 | 273, 255, 161 |
| **47** | 14.87 | [M-H]- | 329.228 | Hydroxyoctadecanedioc acid | C27H43NO2 | C27H43NO2 | 171, 139 |
| **48** | 15.28 | [M+H]+ | 416.345 | Tomatidine | Tomati I | C27H45NO2 | 273, 255, 163 |
| **49** | 15.99 | [M+H]+ | 416.347 | Tomatidine | Tomati II | C27H45NO2 | 273, 255, 163 |
| **50** | 16.08 | [M-H]- | 329.229 | Hydroxyoctadecanedioc acid | C27H43NO2 | C18H33O5 | 171, 139 |
| **51** | 16.09 | [M+H]+ | 353.225 | Tomatidenol | Tomato II | C27H43NO2 | 414, 369 |
| **52** | 16.49 | [M-H]- | 329.229 | Hydroxyoctadecanedioc acid | C27H43NO2 | C18H33O5 | 171, 139 |
| **53** | 20.54 | [M-H]- | 474.258 | Feruloytyramine glycoside | F-tyr glyc I | C24H29NO9 | 312, 178 |
| **54** | 20.99 | [M-H]- | 474.26 | Feruloytyramine glycoside | F-tyr glyc II | C24H29NO9 | 312, 178 |
| **55** | 22.06 | [M-H]- | 504.305 | Feruloyl-3-methoxytyramine glycoside | F-met-tyr glyc I | C25H31NO10 | 342, 178 |
| **56** | 22.51 | [M-H]- | 504.307 | Feruloyl-3-methoxytyramine glycoside | F-met-tyr glyc II | C25H31NO10 | 342, 178 |

Note: The table only shows the identified metabolites in the various tissue. For differential reprogramming and tissue-specific metabolites, please refer to the correlation analysis **(Figure 5)** and VIP score-plots **(Figure 6).**


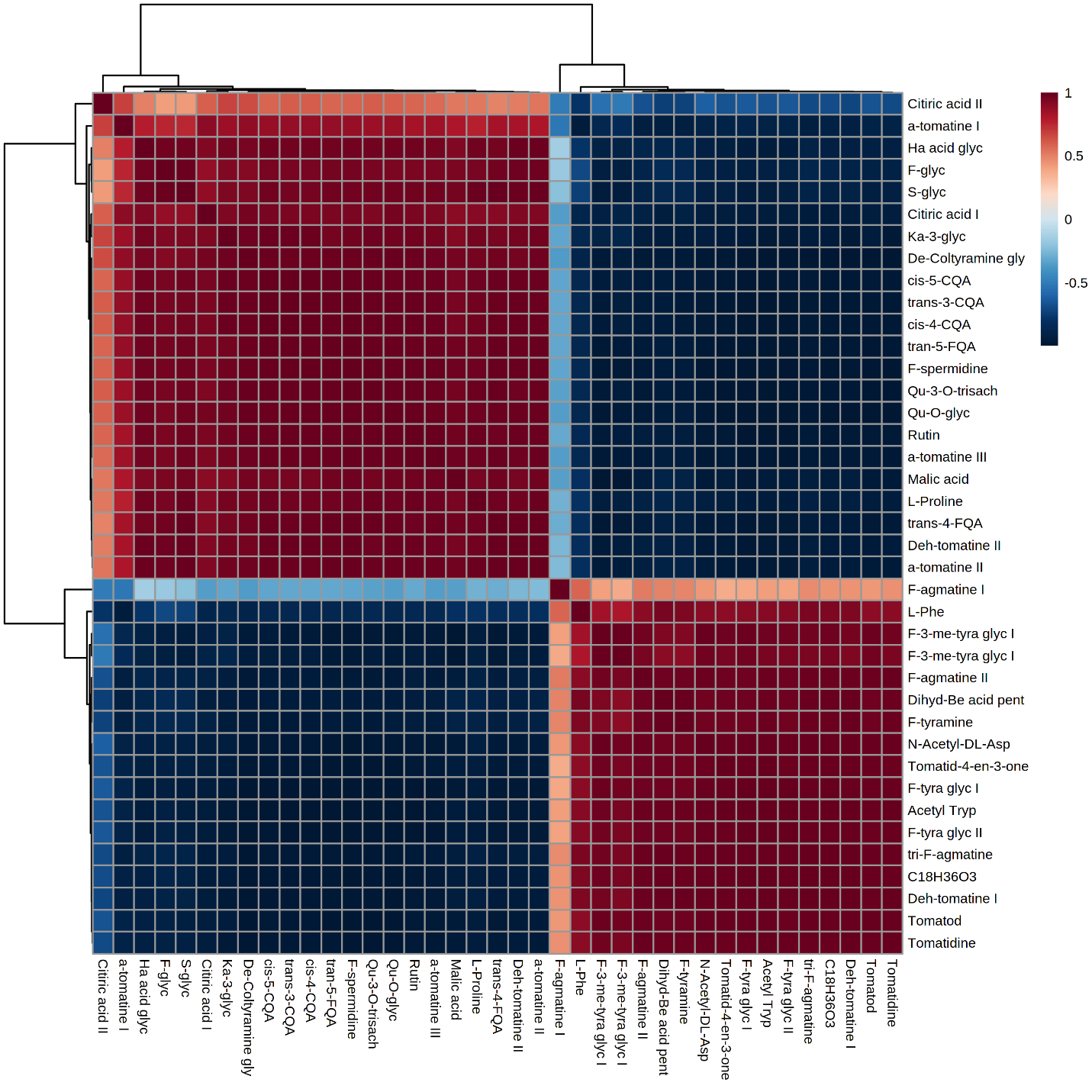


**Figure S16: Correlation matrix among the changes (Δ) within/between extracts from stems of non-treated (NT) day 8 tomato plants and *Phytophthora capsici* (PC)-treated day 6 plants**. Metabolite-metabolite correlations among identified molecules were obtained by deriving a Pearson correlation coefficient. Red indicates a positive correlation, and blue indicates a negative correlation. Abbreviations are explained in **Table S1**. Dendrograms are shown on the top and left of the correlation, indicating clustering of positive and negative correlations.


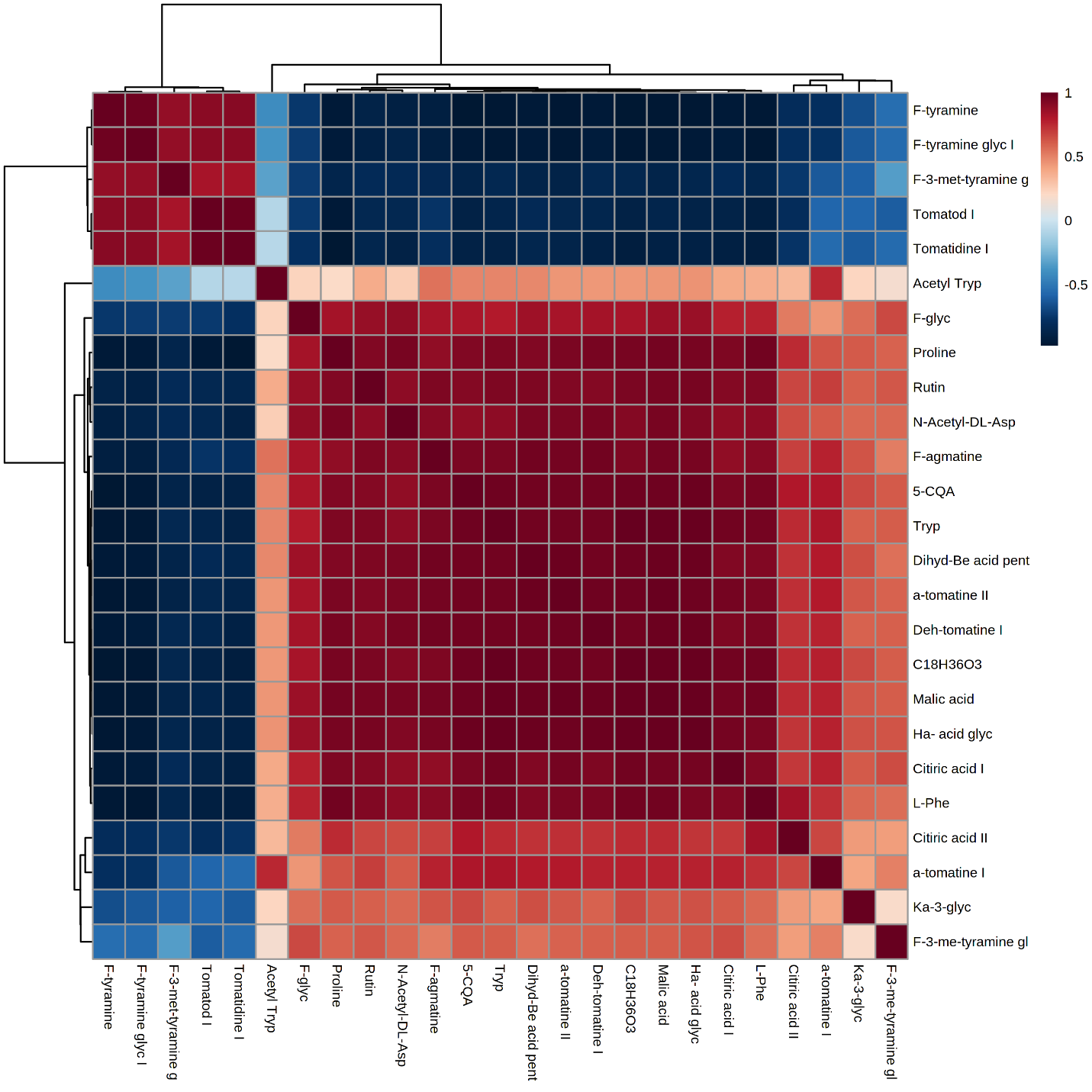


**Figure S17: Correlation matrix among the changes (Δ) within/between extracts from roots of non-treated (NT) day 8 tomato plants and *Phytophthora capsici* (PC)-treated day 6 plants**. Metabolite-metabolite correlations among identified molecules were obtained by deriving a Pearson correlation coefficient. Red indicates a positive correlation, and blue indicates a negative correlation. Abbreviations are explained in **Table S1**. Dendrograms are shown on the top and left of the correlation, indicating clustering of positive and negative correlations.

**Table S2: One-way ANOVA comparing mean values of quantified aromatic amino acids and phytohormones in various tissue of tomato plants infected with *Phytophthora capsici*.**

| **Compound** | ***p*-value** | |
| --- | --- | --- |
| **Roots** | | |
| Phe | | 0.000 |
| Trp | | 0.000 |
| Tyr | | 0.000 |
| MeSA | | 0.000 |
| ACC | | 0.000 |
| **Stems** | | |
| Phe | | 0.000 |
| Trp | | 0.000 |
| Tyr | | 0.000 |
| MeSA | | 0.000 |
| ACC | | 0.000 |
| **Leaves** | | |
| Phe | | 0.000 |
| Trp | | 0.000 |
| Tyr | | 0.000 |
| MeSA | | 0.000 |
| ACC | | 0.000 |

**Table S3: Post-hoc tests comparing mean values of quantified aromatic amino acids and phytohormones in various tissue of tomato plants infected with *Phytophthora capsici*.**

| Compound name | (I) Treatment | (J) Treatment | | *p*-value |
| --- | --- | --- | --- | --- |
|  |  |  |  |  |
| **Roots** | | | | |
| Phe | NT Day 2 | NT Day 8 | | 0.000 |
|  |  | PC Day 2 | | 0.000 |
|  |  | PC Day 4 | | 0.000 |
|  |  | PC Day 6 | | 0.000 |
|  |  | PC Day 8 | | 0.000 |
|  | NT Day 8 | NT Day 2 | | 0.000 |
|  |  | PC Day 2 | | 0.000 |
|  |  | PC Day 4 | | 0.000 |
|  |  | PC Day 6 | | 0.000 |
|  |  | PC Day 8 | | 0.000 |
| Trp | NT Day 2 | NT Day 8 | | 0.013 |
|  |  | PC Day 2 | | 0.000 |
|  |  | PC Day 4 | | 0.000 |
|  |  | PC Day 6 | | 0.000 |
|  |  | PC Day 8 | | 0.005 |
|  | NT Day 8 | NT Day 2 | | 0.013 |
|  |  | PC Day 2 | | 0.000 |
|  |  | PC Day 4 | | 0.000 |
|  |  | PC Day 6 | | 0.000 |
|  |  | PC Day 8 | | 0.999 |
| Tyr | NT Day 2 | NT Day 8 | | 0.914 |
|  |  | PC Day 2 | | 0.000 |
|  |  | PC Day 4 | | 0.000 |
|  |  | PC Day 6 | | 0.000 |
|  |  | PC Day 8 | | 0.000 |
|  | NT Day 8 | NT Day 2 | | 0.914 |
|  |  | PC Day 2 | | 0.000 |
|  |  | PC Day 4 | | 0.000 |
|  |  | PC Day 6 | | 0.000 |
|  |  | PC Day 8 | | 0.000 |
| MeSA | NT Day 2 | NT Day 8 | | 0.510 |
|  |  | PC Day 2 | | 0.004 |
|  |  | PC Day 4 | | 0.000 |
|  |  | PC Day 6 | | 0.198 |
|  |  | PC Day 8 | | 0.001 |
|  | NT Day 8 | NT Day 2 | | 0.510 |
|  |  | PC Day 2 | | 0.000 |
|  |  | PC Day 4 | | 0.000 |
|  |  | PC Day 6 | | 0.002 |
|  |  | PC Day 8 | | 0.122 |
| ACC | NT Day 2 | NT Day 8 | | 0.302 |
|  |  | PC Day 2 | | 0.000 |
|  |  | PC Day 4 | | 0.000 |
|  |  | PC Day 6 | | 0.000 |
|  |  | PC Day 8 | | 0.000 |
|  | NT Day 8 | NT Day 2 | | 0.302 |
|  |  | PC Day 2 | | 0.001 |
|  |  | PC Day 4 | | 0.000 |
|  |  | PC Day 6 | | 0.000 |
|  |  | PC Day 8 | | 0.000 |
| **Stems** | | | | |
| Phe | NT Day 2 | NT Day 8 | | 1.000 |
|  |  | PC Day 2 | | 0.010 |
|  |  | PC Day 4 | | 0.000 |
|  |  | PC day 6 | | 0.000 |
|  |  | PC Day 8 | | 0.000 |
|  | NT Day 8 | NT Day 2 | | 1.000 |
|  |  | PC Day 2 | | 0.022 |
|  |  | PC Day 4 | | 0.000 |
|  |  | PC day 6 | | 0.000 |
|  |  | PC Day 8 | | 0.000 |
| Trp | NT Day 2 | NT Day 8 | | 0.999 |
|  |  | PC Day 2 | | 0.666 |
|  |  | PC Day 4 | | 0.000 |
|  |  | PC day 6 | | 0.000 |
|  |  | PC Day 8 | | 0.000 |
|  | NT Day 8 | NT Day 2 | | 0.999 |
|  |  | PC Day 2 | | 0.862 |
|  |  | PC Day 4 | | 0.000 |
|  |  | PC Day 6 | | 0.000 |
|  |  | PC Day 8 | | 0.000 |
| Tyr | NT Day 2 | NT Day 8 | | 0.908 |
|  |  | PC Day 2 | | 0.008 |
|  |  | PC Day 4 | | 0.000 |
|  |  | PC Day 6 | | 0.000 |
|  |  | PC Day 8 | | 0.000 |
|  | NT Day 8 | NT Day 2 | | 0.908 |
|  |  | PC Day 2 | | 0.108 |
|  |  | PC Day 4 | | 0.000 |
|  |  | PC Day 6 | | 0.000 |
|  |  | PC Day 8 | | 0.000 |
| MeSA | NT Day 2 | NT Day 8 | | 0.810 |
|  |  | PC Day 2 | | 0.001 |
|  |  | PC Day 4 | | 0.002 |
|  |  | PC Day 6 | | 0.888 |
|  |  | PC Day 8 | | 0.000 |
|  | NT Day 8 | NT Day 2 | | 0.810 |
|  |  | PC Day 2 | | 0.025 |
|  |  | PC Day 4 | | 0.059 |
|  |  | PC Day 6 | | 1.000 |
|  |  | PC Day 8 | | 0.001 |
| ACC | NT Day 2 | | NT Day 8 | 0.994 |
|  |  |  | PC Day 2 | 0.939 |
|  |  |  | PC Day 4 | 0.000 |
|  |  |  | PC Day 6 | 0.000 |
|  |  |  | PC Day 8 | 0.000 |
|  | NT Day 8 | | NT Day 2 | 0.994 |
|  |  |  | PC Day 2 | 0.683 |
|  |  |  | PC Day 4 | 0.000 |
|  |  |  | PC Day 6 | 0.000 |
|  |  |  | PC Day 8 | 0.000 |
| **Leaves** | | | | |
| Phe | NT Day 2 | | NT Day 8 | 1.000 |
|  |  |  | PC Day 2 | 0.000 |
|  |  |  | PC Day 4 | 0.000 |
|  |  |  | PC Day 6 | 0.000 |
|  |  |  | PC Day 8 | 0.000 |
|  | NT Day 8 | | NT Day 2 | 1.000 |
|  |  |  | PC Day 2 | 0.000 |
|  |  |  | PC Day 4 | 0.000 |
|  |  |  | PC Day 6 | 0.000 |
|  |  |  | PC Day 8 | 0.000 |
|  | PC Day 2 | | NT Day 2 | 0.000 |
|  |  |  | NT Day 8 | 0.000 |
|  |  |  | PC Day 4 | 0.000 |
|  |  |  | PC Day 6 | 0.000 |
|  |  |  | PC Day 8 | 0.000 |
| Trp | NT Day 2 | | NT Day 8 | 0.993 |
|  |  |  | PC Day 2 | 0.000 |
|  |  |  | PC Day 4 | 0.000 |
|  |  |  | PC Day 6 | 0.000 |
|  |  |  | PC Day 8 | 0.000 |
|  | NT Day 8 | | NT Day 2 | 0.993 |
|  |  |  | PC Day 2 | 0.000 |
|  |  |  | PC Day 4 | 0.000 |
|  |  |  | PC Day 6 | 0.000 |
|  |  |  | PC Day 8 | 0.000 |
| Tyr | NT Day 2 | | NT Day 8 | 1.000 |
|  |  |  | PC Day 2 | 0.395 |
|  |  |  | PC Day 4 | 0.000 |
|  |  |  | PC Day 6 | 0.000 |
|  |  |  | PC Day 8 | 0.000 |
|  | NT Day 8 | | NT Day 2 | 1.000 |
|  |  |  | PC Day 2 | 0.379 |
|  |  |  | PC Day 4 | 0.000 |
|  |  |  | PC Day 6 | 0.000 |
|  |  |  | PC Day 8 | 0.000 |
| MeSA | NT Day 2 | | NT Day 8 | 0.946 |
|  |  |  | PC Day 2 | 0.747 |
|  |  |  | PC Day 4 | 0.390 |
|  |  |  | PC Day 6 | 0.449 |
|  |  |  | PC Day 8 | 0.110 |
|  | NT Day 8 | | NT Day 2 | 0.946 |
|  |  |  | PC Day 2 | 0.997 |
|  |  |  | PC Day 4 | 0.900 |
|  |  |  | PC Day 6 | 0.086 |
|  |  |  | PC Day 8 | 0.011 |
| ACC | NT Day 2 | | NT Day 8 | 0.933 |
|  |  |  | PC Day 2 | 1.000 |
|  |  |  | PC Day 4 | 0.000 |
|  |  |  | PC Day 6 | 0.000 |
|  |  |  | PC Day 8 | 0.000 |
|  | NT Day 8 | | NT Day 2 | 0.933 |
|  |  |  | PC Day 2 | 0.856 |
|  |  |  | PC Day 4 | 0.000 |
|  |  |  | PC Day 6 | 0.000 |
|  |  |  | PC Day 8 | 0.000 |
